# Endoplasmic reticulum stress-related super enhancer promotes epithelial-mesenchymal transformation in hepatocellular carcinoma through CREB5 mediated activation of TNC

**DOI:** 10.1038/s41419-025-07356-y

**Published:** 2025-02-06

**Authors:** Anqi Wang, Sitong Yan, Jiatao Liu, Xiang Chen, Mengyao Hu, Xiao Du, Weijia Jiang, Zhipeng Pan, Lulu Fan, Guoping Sun

**Affiliations:** 1 Department of Oncology, The First Affiliated Hospital of Anhui Medical University, Hefei, Anhui China; 2 Department of Integrated Traditional Chinese and Western Medicine, The First Affiliated Hospital of Anhui Medical University, Hefei, Anhui China; 3 Department of Pharmacy, The First Affiliated Hospital of Anhui Medical University, Hefei, Anhui China; 4 Department of Oncology, The Second Hospital of Anhui Medical University, Hefei, Anhui China

**Keywords:** Oncogenes, Metastasis, Liver cancer

## Abstract

Super-enhancers (SEs) are associated with key genes that control cellular state and cell identity. Endoplasmic reticulum stress (ERS) regulates epithelial-mesenchymal transformation (EMT). However, whether SEs are involved in ERS-related activation of EMT in hepatocellular carcinoma (HCC) is unknown. In this study, we identified 17 ERS-related SEs by comparing ERS-HCC cells with untreated control cells using ChIP-seq and RNA-seq. CRISPR-Cas9 and RT-qPCR identified CAMP responsive element binding protein 5 (CREB5) as a key target of ERS-related SE. Analyses of TCGA datasets and tissue arrays showed that CREB5 mRNA and protein expression levels were higher in liver cancer tissues than in paired normal tissues. In addition, overexpression of CREB5 was associated with poor prognosis and an aggressive phenotype in patients with HCC. We also found that activation of ERS enhanced the expression of CREB5, and upregulation of CREB5 significantly increased cell proliferation, migration, and invasion, and promoted EMT, but inhibited apoptosis. More importantly, ERS activation increased the expression of several EMT markers by modulating the expression of CREB5. Mechanistically, CREB5 upregulates the transcription of tenascin-C (TNC) by directly binding to its promoter region, thereby promoting EMT in liver cancer cells. In summary, our findings suggest that ERS activation promotes EMT in liver cancer cells via SE-mediated upregulation of the CREB5/TNC pathway. This result provides a new direction for uncovering how ERS regulates EMT and a foundation for preventing the progression of EMT in HCC.

## Introduction

Hepatocellular carcinoma (HCC) is a prevalent malignancy with poor long-term prognosis due to its insidious onset, high recurrence and metastasis rate [1, 2]. Therefore, further understanding of the molecular mechanisms underlying liver cancer tumor growth and metastasis is critical.

Endoplasmic reticulum stress (ERS) arises from the accumulation of unfolded or misfolded proteins in the endoplasmic reticulum lumen and triggers an adaptive response known as the unfolded protein response (UPR) [3]. Our previous studies showed that ERS promotes the development of liver cancer by enhancing angiogenesis [4], inducing autophagy [5], and decreasing the sensitivity of liver cancer cells to chemotherapeutic agents [6, 7]. Emerging evidence suggests that ERS and the UPR pathway are closely linked to the activation of epithelial-mesenchymal transformation (EMT) in several cancers [8, 9]. For instance, activation of the PERK-eIF2α pathway was shown to be essential for EMT and the invasive properties of cancer cells [10]. Similarly, the IRE1α and ATF6 pathways of the UPR have been implicated in the regulation of EMT marker expression and cell migration [11, 12]. The role of ERS in regulating EMT is further underscored by its role in modulating the expression of EMT-related proteins, such as the epithelial marker E-cadherin and the mesenchymal markers N-cadherin and vimentin [13]. Understanding the mechanisms linking ERS to EMT could reveal new therapeutic strategies; however, how ERS affects EMT in liver cancer is not fully understood.

Super enhancers (SEs) are long cis-acting elements of 8–20 kb length with transcription-enhancing activity. They enrich high-density histone modification marks, master transcription factors, and cofactors [14]. Many key oncogenes in tumor cells are driven by SEs and they are important for regulating EMT in liver cancer via binding to specific transcription factors [15, 16]. However, the role of ERS-related SEs and their target genes in HCC development and EMT remains to be clarified.

CAMP responsive element binding protein 5 (CREB5) is a transcription factor and has been shown to play a key role in the development and progression of various cancers [17–19]. Studies have shown that CREB5 expression is upregulated in HCC, and its high expression is associated with poor prognosis [20] and malignant progression [21, 22]. However, the specific mechanism of CREB5 involvement in EMT in HCC cells, and the relationship between CREB5-related pathways and ERS in HCC remain unclear.

In this study, we identified ERS-related SEs and their key target gene in liver cancer cells using RNA-seq and ChIP-seq. Further experiments demonstrated that CREB5 was regulated by ERS and ERS-related SE. CREB5 directly bound to the tenascin-C (TNC) promoter to induce EMT in liver cancer cells, which ultimately enhanced invasion and metastasis. Our results further expand the relationship among ERS, SE, and EMT in promoting HCC progression, and provide a basis for targeting SE and related genes for HCC treatment.

## Results

### CREB5 is a key target gene of ERS-related SE in HCC

**Fig. 1:**
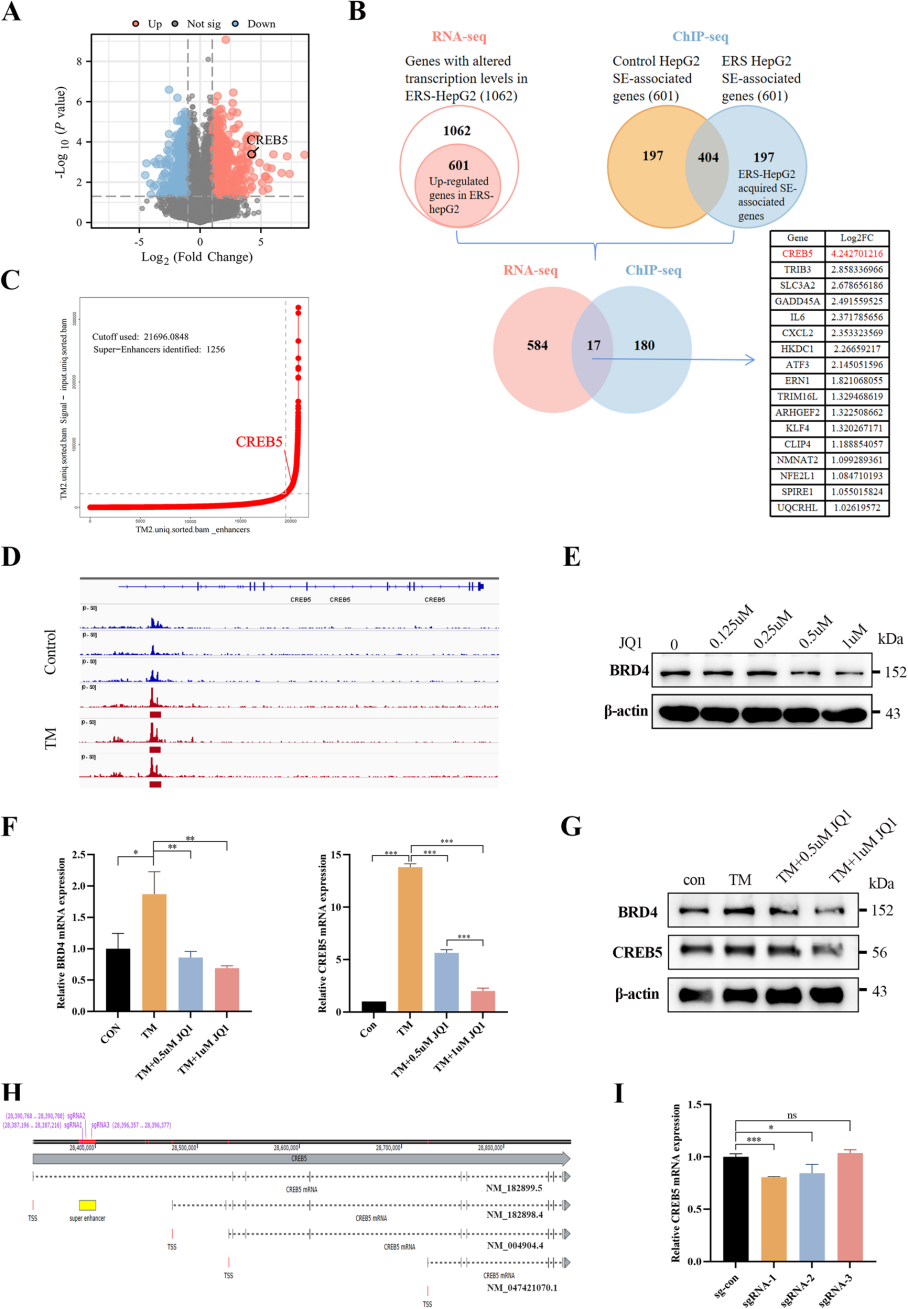
CREB5 is a key target gene of ERS-related SE in HCC. **A** RNA sequencing volcano mapping of TM treated or untreated HepG2 cells. **B** The Venn diagram of differentially expressed genes in TM treated or untreated HepG2 cells analyzed by RNA-seq and ChIP-seq. **C** Hockey stick plots on the basis of their input-normalized H3K27ac signals in the TM treated HepG2 cells; CREB5 is highlighted. **D** H3K27ac signal was significantly enriched in ERS-HepG2 cells compared with that in normal HepG2 cells. **E** Changes in BRD4 protein levels in HepG2 cells treated with different concentrations of JQ1. Changes in the mRNA (**F**) and protein (**G**) levels of BRD4 and CREB5 in HepG2 cells treated with 2.5 µM TM and different concentrations of JQ1. **H** Three sgRNAs were designed to target specific regions of SEs. **I** The blockade of three individual constituent sites on the SEs affects CREB5 transcription levels.

To determine the roles of ERS activation and SE in the invasion and migration of liver cancer, we first performed RNA-seq on tunicamycin (TM)-treated and untreated HepG2 cells and found 1062 differentially expressed genes (DEGs) in TM-induced ERS HepG2 cells (Fig. 1A), including 601 upregulated genes (Log_2_FC ≥ 1.0, *p* < 0.05). Subsequently, we used ChIP-seq analysis to detect changes in SEs in HepG2 cells with TM-induced ERS and found significant changes in 197 ERS-specific SEs. Finally, we used an online tool (http://www.ehbio.com/test/venn/#/) to identify potential target genes of ERS-related SEs, which identified 17 genes (Fig. 1B). We analyzed the expression of these 17 genes in HCC and normal liver tissue using The Cancer Genome Atlas Liver Hepatocellular Carcinoma (TCGA-LIHC) dataset (Supplementary Fig. 1A). Gene ontology (GO) and Kyoto Encyclopedia of Genes and Genomes (KEGG) analyses were performed on these 17 target genes, which showed that they are widely involved in ERS, cell adhesion, angiogenesis, and other biological processes closely related to tumor occurrence and metastasis (Supplementary Fig. 1B). We selected CREB5, the gene with the highest fold change (Log_2_FC = 4.243, *p* < 0.05), for further analyses. In the ChIP-seq assay, SE-associated CREB5 was detected in ERS-HepG2 cells (Fig. 1C), and the H3K27ac signaling was significantly higher in the upstream non-coding genomic region of CREB5 in ERS-HepG2 cells than in normal HepG2 cells (Fig. 1D). JQ1, an inhibitor of BRD4, effectively disrupts SE activity in various cancers [23–25]. We treated HepG2 cells with different concentrations of JQ1, and western blotting showed that 0.5 and 1 μM JQ1 effectively inhibited BRD4 expression (Fig. 1E). In addition, JQ1 increased the mRNA expression of the epithelial marker E-cadherin, but decreased the mesenchymal markers N-cadherin and vimentin (Supplementary Fig. 1C). Moreover, JQ1 treatment effectively inhibited the expression of BRD4 and CREB5 at both mRNA and protein levels under ERS (Fig. 1F, G). To further determine the functional region of CREB5-SE, we designed three sgRNAs based on the peaks showing significant enrichment of the H3K27ac signal in ERS-HepG2 cells using CRISPR interference and dCas9-KRAB to interfere with SE-promoter interactions (Fig. 1H). RT-qPCR analysis showed that sgRNA-1 and sgRNA-2 reduced CREB5 transcript levels, whereas sgRNA-3 caused no significant changes (Fig. 1I).

### CREB5 is highly expressed in HCC and associated with poor prognosis

**Fig. 2:**
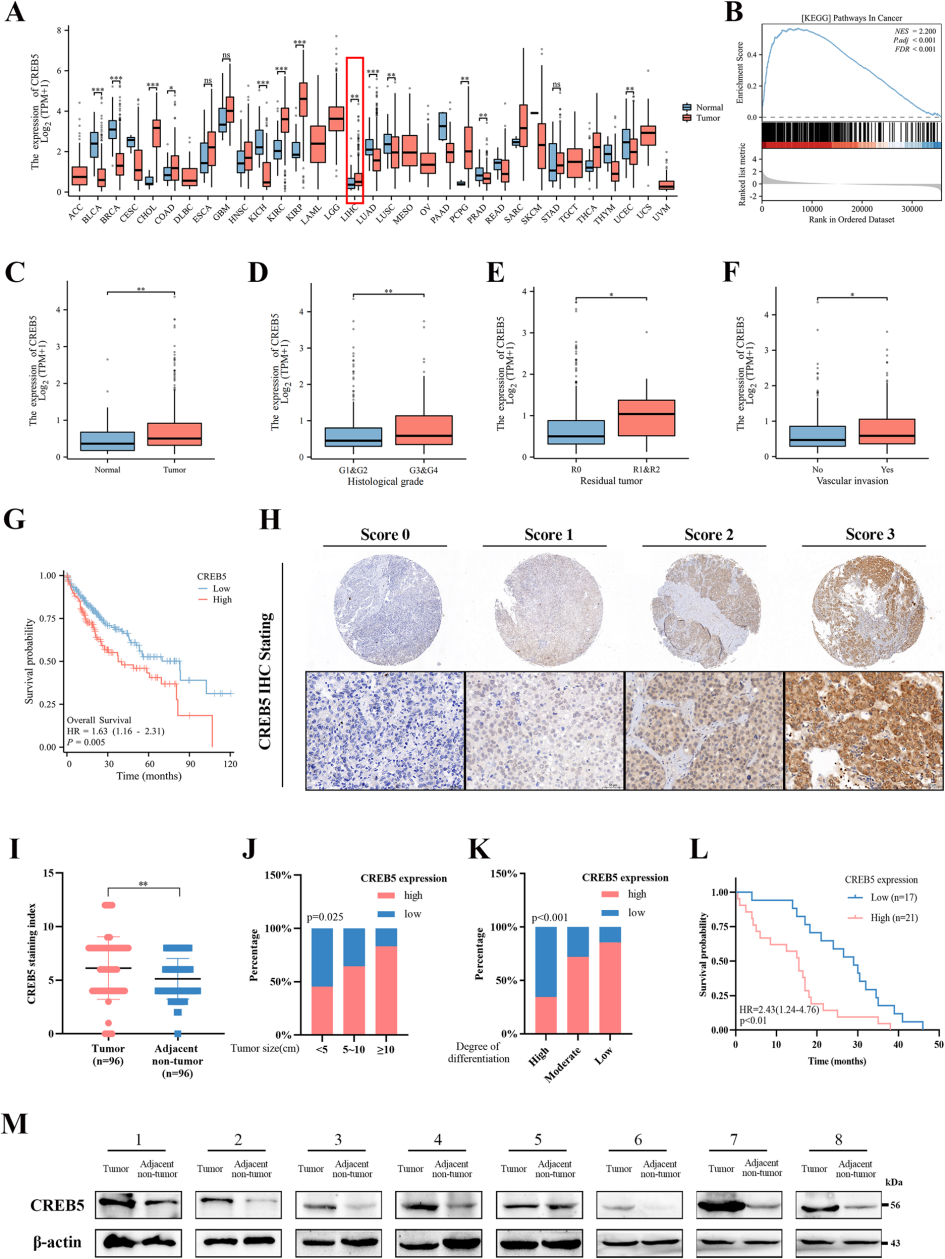
CREB5 is highly expressed in HCC and associated with poor prognosis. **A** The expression of CREB5 in 33 different cancer tissues and adjacent tissues was analyzed by TCGA database. **B** GSEA maps showing that tumor-related gene sets were significantly enriched in the high CREB5 expression group. **C** Expression profile of CREB5 mRNA in HCC tissues and normal tissues (TCGA). **D** CREB5 expression in different histological grade gruops (TCGA). Effect of CREB5 expression on residual tumor (**E**) and vascular invasion (**F**) in HCC (TCGA). **G** Kaplan–Meier survival curves of OS based on TCGA database. **H** Representative images of IHC staining intensity levels of 0, 1, 2 and 3. **I** IHC staining of CREB5 in liver cancer and adjacent tissue. IHC staining of CREB5 in the different tumor sizes (**J**) and histological grades (**K**). **L** Kaplan–Meier analysis of OS stratified by high CREB5 expression (*n* = 21) and low CREB5 expression (*n* = 17). **M** The image of the relative levels of CREB5 in liver cancer tissues and adjacent non-tumor liver tissue by western-blotting.

According to TCGA datasets, CREB5 was highly expressed in various tumor tissues (Fig. 2A). Gene set enrichment analysis (GSEA) revealed significant enrichment of tumor-related genes in the CREB5 high-expression group (NES = 2.20, p.adj < 0.001) (Fig. 2B). Analysis of the TCGA-LIHC dataset showed significantly higher levels of CREB5 in HCC tissues than in normal tissues (Fig. 2C). CREB5 expression was also associated with histological grade (Fig. 2D), residual cancer (Fig. 2E), and vascular invasion (Fig. 2F). Moreover, survival analysis showed that high expression of CREB5 reduced OS (*p* = 0.005) (Fig. 2G) and disease-specific survival (*p* = 0.045) (Supplementary Fig. 1D), but had no effect on progress free interval (*p* > 0.05) (Supplementary Fig. 1E). IHC staining was performed on microarrays containing tumor and adjacent normal tissue samples from 96 patients with HCC. Tumors were divided into four grades (0, 1, 2, and 3) according to the staining intensity of CREB5 (Fig. 2H). The results showed that CREB5 expression was higher in tumors than in adjacent normal tissues (Fig. 2I). We also explored the relationship between CREB5 expression and the clinicopathological features of HCC, finding that high CREB5 expression was correlated with tumor size (Fig. 2J) and degree of differentiation (Fig. 2K), but not with age, sex, history of hepatitis/cirrhosis, or AFP level (Supplementary Table 1). We also tracked the OS of 38 HCC patients and patients with higher CREB5 expression had a poorer prognosis (*p* < 0.01) (Fig. 2L). Furthermore, we collected eight pairs of freshly resected liver cancer tissues and adjacent liver tissues for western blotting of CREB5, which showed significantly higher CREB5 expression in HCC tissues than in adjacent normal liver tissues (Fig. 2M).

### Activation of ERS upregulates CREB5 expression in HCC

**Fig. 3:**
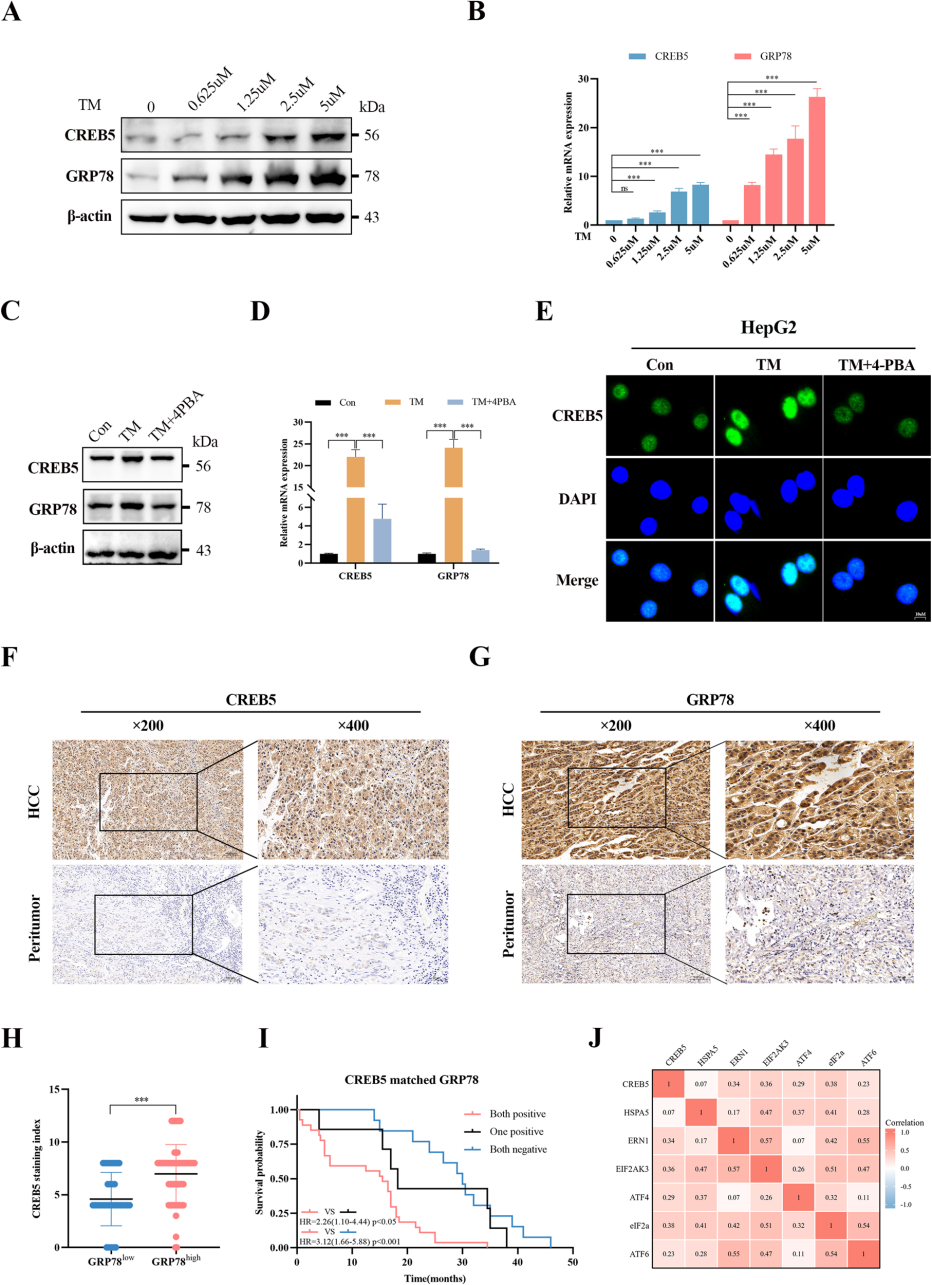
Activation of ERS upregulates CREB5 expression in HCC. Changes in GRP78 and CREB5 protein (**A**) and mRNA (**B**) levels after HepG2 cells were treated with different concentrations of TM. Changes in GRP78 and CREB5 protein (**C**) and mRNA (**D**) levels after HepG2 cells were treated with TM (2.5 µM) or TM (2.5 µM) + 4-PBA (1 mM). **E** HepG2 cells were treated with TM (2.5 µM) or TM (2.5 µM) + 4-PBA (1 mM), and the expression of CREB5 was detected by immunofluorescence. IHC staining images of CREB5 (**F**) and GRP78 (**G**) in liver cancer tissue and adjacent normal liver tissue. **H** IHC staining index of CREB5 in the GRP78 high/low group. **I** Kaplan–Meier survival curves of OS in related groups. **J** The correlation between CREB5 and ERS-related downstream genes was analyzed through the mRNA expression of 424 cases with HCC in TCGA database.

To explore whether ERS promotes the progression of HCC via modulating the expression of CREB5, HepG2 cells were treated with a series of concentrations of TM. Western blotting and RT-qPCR results showed that both CREB5 and glucose-regulated protein 78 (GRP78) were increased by TM in a dose-dependent manner (Fig. 3A, B). We also treated HepG2 cells with the ERS inhibitor 4-phenylbutyric acid (4-PBA, 1 mM). Treatment with 4-PBA inhibited TM-induced upregulation of CREB5 and GRP78 (Fig. 3C, D). Immunofluorescence assays showed that CREB5 expression was significantly increased after TM treatment and that CREB5 localized to the nucleus, whereas 4-PBA significantly reduced CREB5 expression (Fig. 3E, Supplementary Fig. 1F). We also performed IHC staining of CREB5 and GRP78 on 96 pairs of HCC and adjacent normal liver tissues, revealing that both CREB5 and GRP78 were highly expressed in HCC tissues compared with levels in normal tissues (Fig. 3F, G). Further, CREB5 expression was positively correlated with GRP78 levels (Fig. 3H) (Supplementary Table 2). More importantly, OS was shorter in CREB5/GRP78-double positive patients (*p* < 0.05) (Fig. 3I). Bioinformatics analysis of TCGA data showed that CREB5 was positively correlated with ERS-related downstream genes (Fig. 3J).

### CREB5 promotes proliferation of HCC in vivo and in vitro

**Fig. 4:**
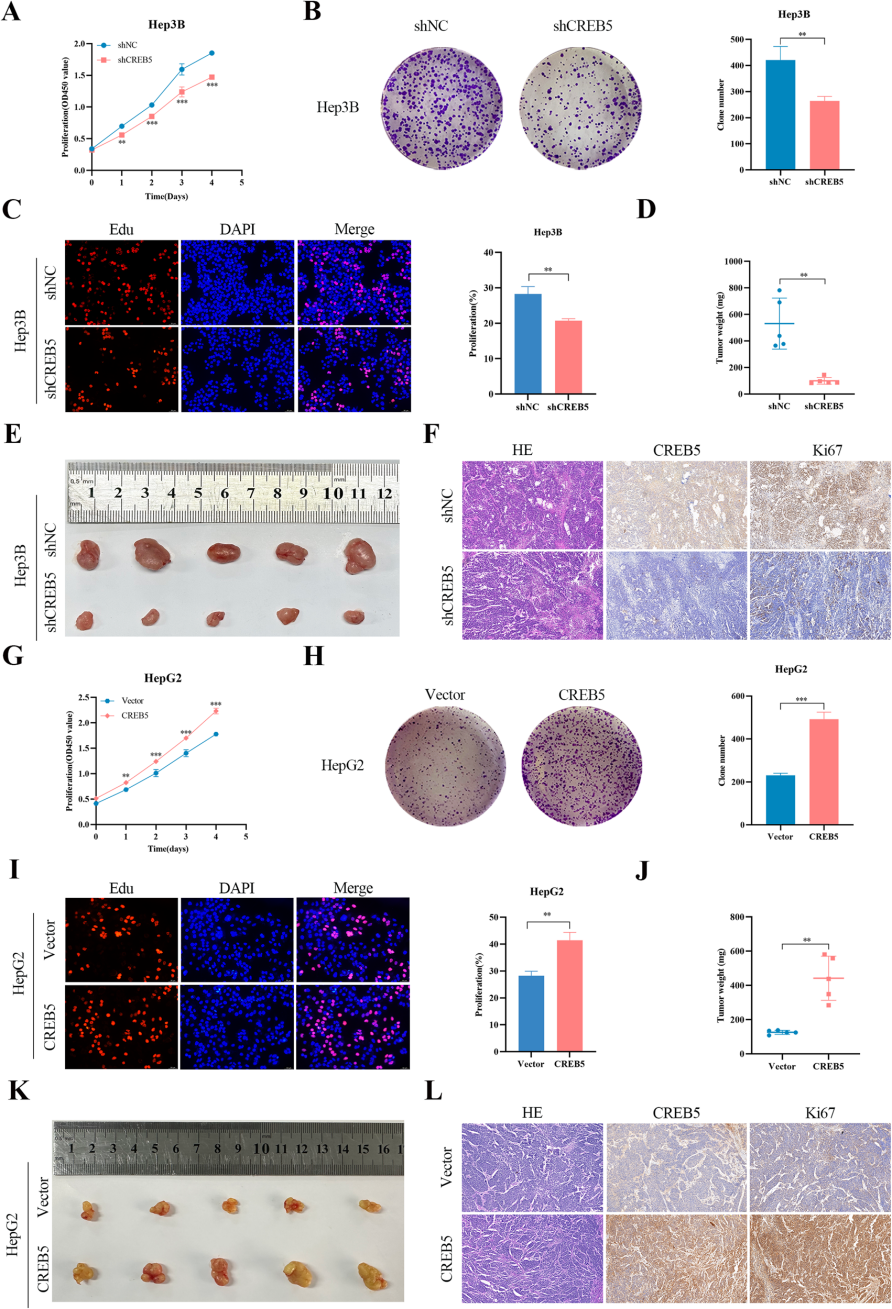
CREB5 promotes proliferation of HCC in vivo and in vitro. The effect of CREB5 on the proliferation of Hep3B cells was detected by CCK-8 assay (**A**), colony formation assay (**B**), and EDU (**C**). Tumors from mice in the shNC and shCREB5 groups (**E**) and tumor weights (**D**) were measured. **F** HE and IHC staining of CREB5 and Ki67 indices was performed on the tumors of the shNC and shCREB5 groups. The effect of CREB5 on the proliferation of HepG2 cells was detected by CCK-8 assay (**G**), colony formation assay (**H**), and EDU (**I**). Tumors from mice in vector and CREB5 groups (**K**) and tumor weights were measured (**J**). **L** HE and IHC staining of CREB5 and Ki67 indices was performed on the tumors of the vector and CREB5 groups.

To further explore the effect of CREB5 on the development of HCC, we analyzed CREB5 expression in several liver cancer cell lines using western blotting and RT-qPCR (Supplementary Fig. 1G). CREB5 expression was relatively high in Hep3B and LM3 cells but was lower in HepG2 and Huh7 cells. We then knocked down CREB5 in Hep3B and LM3 cells (Supplementary Fig. 2A, B) but overexpressed it in HepG2 and Huh7 cells (Supplementary Fig. 2F, G) using CREB5-shRNA and CREB5-overexpression lentiviruses, respectively. The effect of CREB5 expression on cell proliferation was evaluated using CCK-8, colony formation, and EDU proliferation staining assays, which showed that knockdown of CREB5 in Hep3B cells significantly inhibited cell proliferation (Fig. 4A–C). This phenomenon was also confirmed in LM3 cells (Supplementary Fig. 2C–E). Animal experiments showed that CREB5 knockdown significantly reduced tumor weight (Fig. 4D) and volume (Fig. 4E) in a subcutaneous xenograft model using Hep3B cells. IHC also showed that CREB5 knockdown significantly reduced Ki67 expression compared to that in control mice (Fig. 4F). In contrast, overexpression of CREB5 increased the proliferation of HepG2 (Fig. 4G–I) and Huh7 cells (Supplementary Fig. 2H–J) compared to that of the negative controls. In vivo experiments showed that the tumor size and weight were significantly greater in mice that overexpressed CREB5 than those in the vector group (Fig. 4J, K). IHC also showed that Ki67 expression in the CREB5-overexpression group was higher than that in the control group (Fig. 4L).

### CREB5 promotes migration and invasion and inhibits apoptosis of HCC

**Fig. 5:**
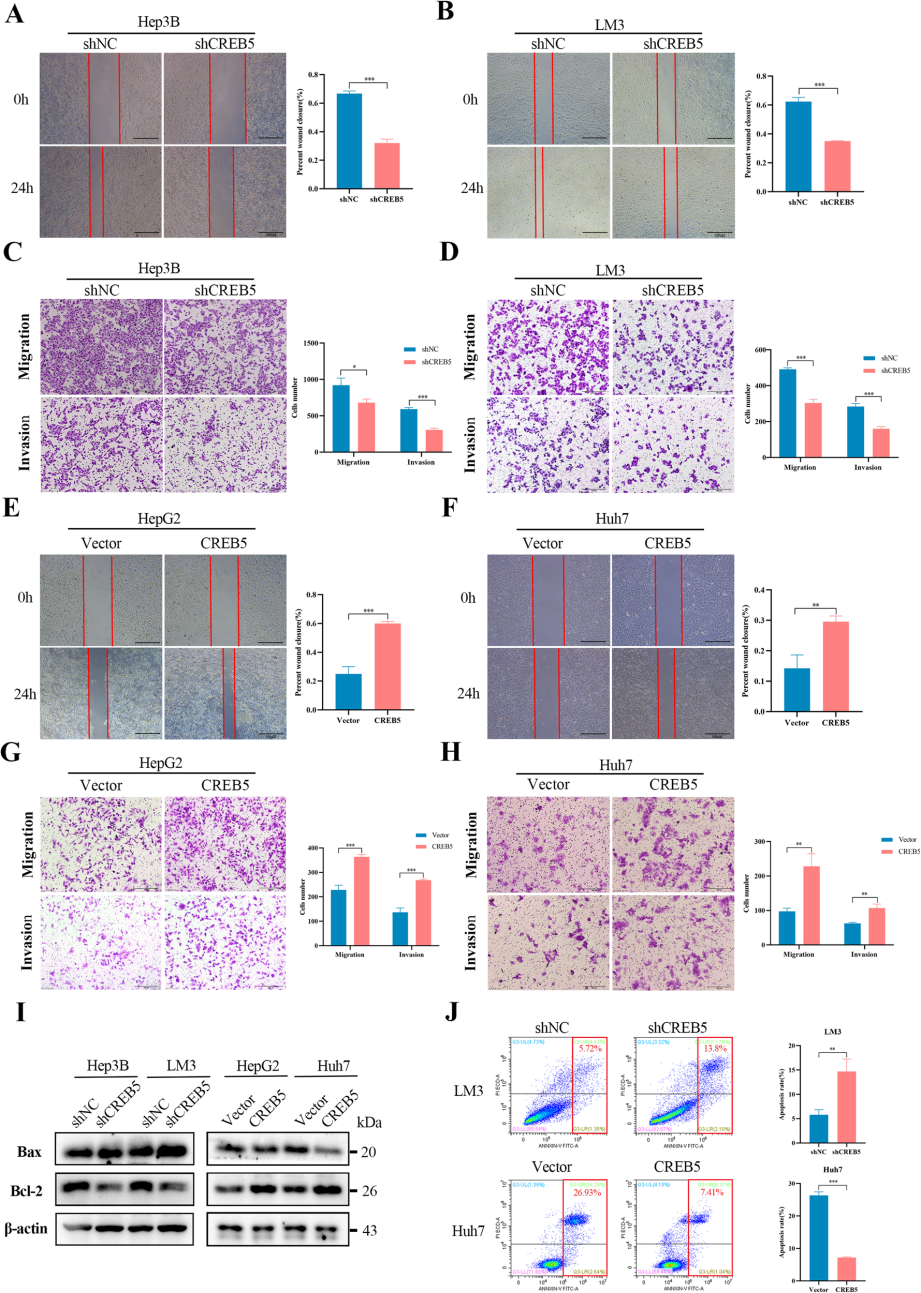
CREB5 promotes migration and invasion and inhibits apoptosis of HCC. The migration ability of Hep3B (**A**) and LM3 (**B**) cells after CREB5 knockdown was examined by wound-healing assay. The migration and invasion ability of Hep3B (**C**) and LM3 (**D**) after CREB5 knockdown was examined by transwell assay. The migration ability of HepG2 (**E**) and Huh7 (**F**) cells after CREB5 overexpression was examined by wound-healing assay. The migration and invasion ability of HepG2 (**G**) and Huh7 (**H**) after CREB5 overexpression was examined by transwell assay. **I** Protein expression of BAX and Bcl-2 after CREB5 knockdown or overexpression. **J** Flow cytometry was used to detect cell apoptosis after CREB5 knockdown or overexpression.

To determine the effect of CREB5 on the migration and invasion of liver cancer cells, wound healing and transwell assays were performed. CREB5 knockdown reduced both cell migration and invasion in Hep3B and LM3 cells (Fig. 5A–D). In contrast, CREB5 overexpression enhanced the migration and invasion of HepG2 and Huh7 cells compared to those of the control cells (Fig. 5E–H). In addition, downregulation of CREB5 significantly decreased the expression of Bcl-2, while increasing the expression of Bax, and promoted apoptosis compared to observations in the shNC group. In contrast, overexpression of CREB5 inhibited apoptosis (Fig. 5I). These results were further confirmed by flow cytometry (Fig. 5J).

### ERS promotes EMT through CREB5

**Fig. 6:**
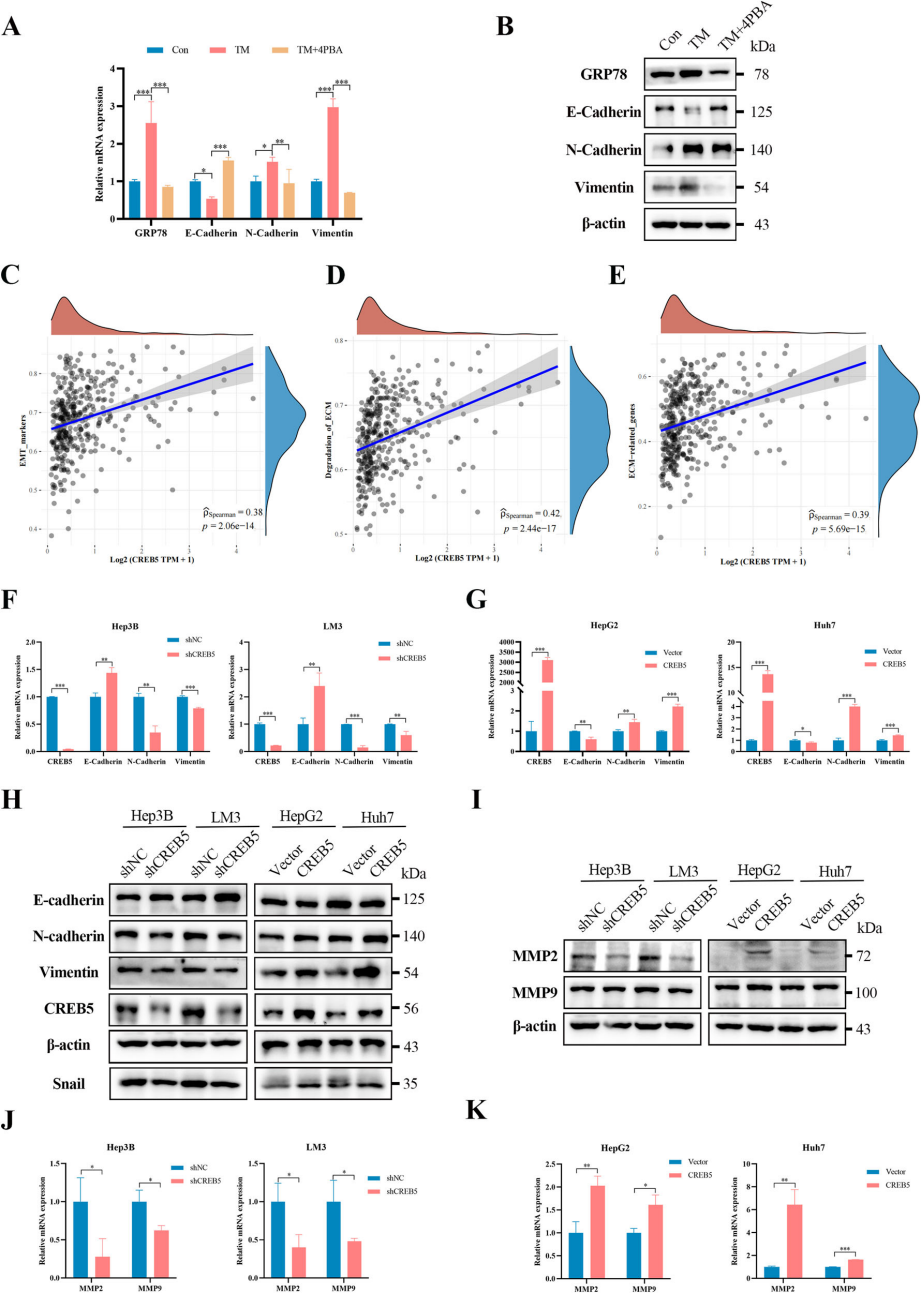
ERS promotes EMT through CREB5. Relative mRNA (**A**) and protein (**B**) expression of ERS markers (GRP78) and EMT markers (E-cadherin, N-cadherin, and vimentin) in HepG2 cells treated with TM (2.5 µM) or TM (2.5 µM) + 4-PBA (1 mM). **C**–**E** Spearman correlation analysis between CREB5 and different pathways (TCGA). The relative mRNA (**F**, **G**) and protein (**H**) expression levels of EMT markers in cells with CREB5 overexpression or knockdown. The relative protein (**I**) and mRNA (**J**, **K**) expression levels of MMP2 and MMP9 in cells with CREB5 overexpression or knockdown.

To test whether ERS promotes EMT in HCC cells by modulating the expression of CREB5, we treated HepG2 cells with TM for 24 h to induce ERS. RT-qPCR and western blotting results show that TM decreased the expression of E-cadherin but increased that of N-cadherin and vimentin, while co-treatment with 4-PBA for 24 h reversed these effects of TM (Fig. 6A, B). Moreover, we analyzed the RNA-seq data in TCGA and collected gene sets from related pathways. Spearman correlation analysis showed that CREB5 was positively correlated with EMT markers (Fig. 6C), degradation of extracellular matrix (ECM) (Fig. 6D), and ECM-related genes (Fig. 6E). GSEA revealed significant associations between CREB5 and ECM receptor interactions (Supplementary Fig. 3A), ECM organization (Supplementary Fig. 3B), matrix metalloproteinases (MMPs) (Supplementary Fig. 3C), and the PI3K-AKT signaling pathway (Supplementary Fig. 3D). In the human liver cancer cell lines Hep3B and LM3, knockdown of CREB5 increased the expression of the epithelial marker E-cadherin but decreased that of the mesenchymal markers N-cadherin and vimentin, as well as the EMT transcription factor Snail. The opposite effects were observed in HepG2 and Huh7 cells overexpressing CREB5 (Fig. 6F–H). MMPs degrade the ECM, and thus promote tumor cell invasion and metastasis [26]. To confirm the correlation between CREB5 and ECM degradation, we measured the expression levels of MMP2 and MMP9 in CREB5-knockdown and -overexpressing cells. The expression levels of both MMP2 and MMP9 were decreased in CREB5 knockdown cells but increased in CREB5-overexpressing cells (Fig. 6I–K). Further, we knocked down or overexpressed CREB5 in a mouse liver cancer cell line Hep1-6 (Supplementary Fig. 3E, F) and found that CREB5 promoted EMT and increased the expression of MMP2 and MMP9 in Hep1-6 cells (Supplementary Fig. 3G–J).

### TNC is a downstream effector of CREB5

**Fig. 7:**
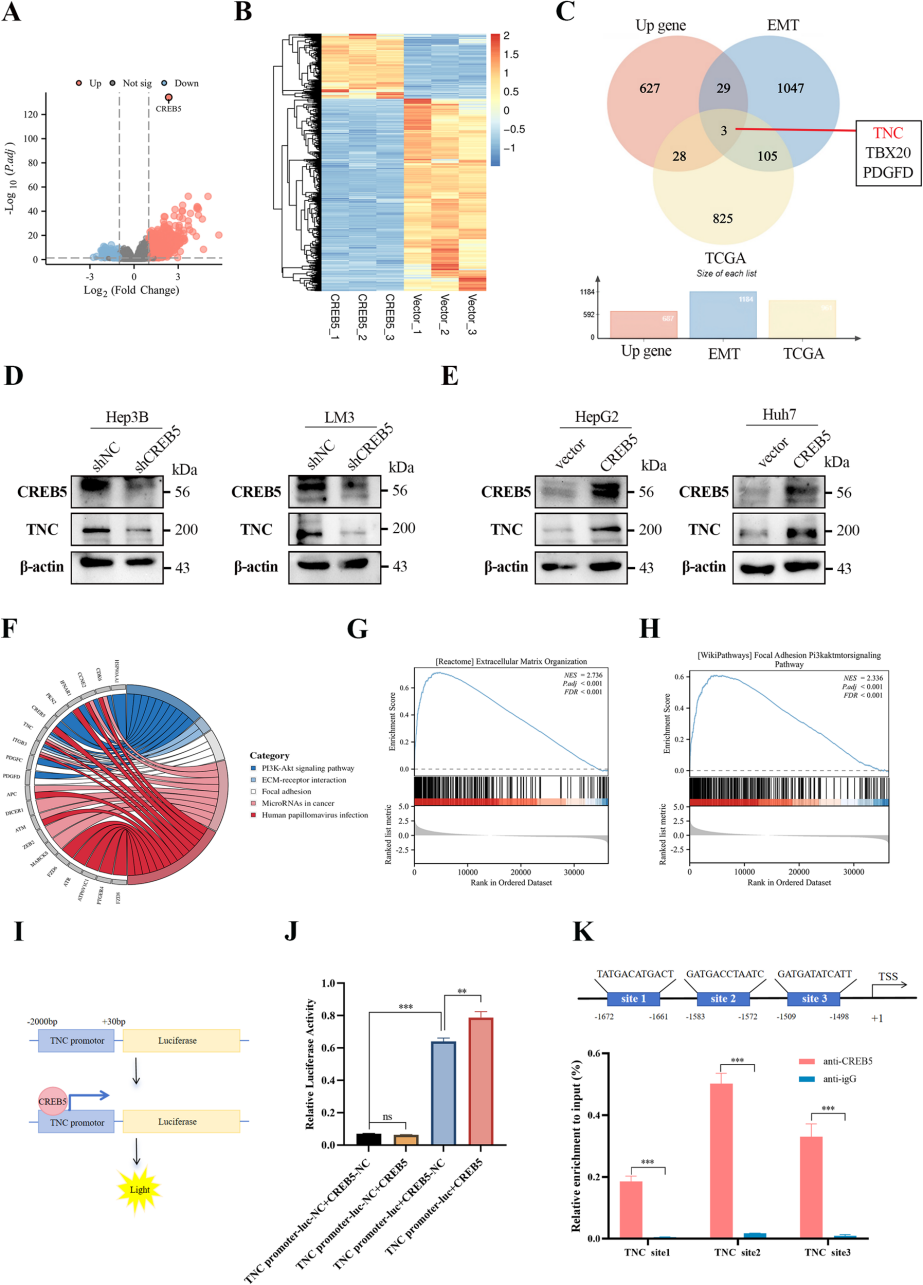
TNC is a downstream effector of CREB5. **A** Single-gene differential analysis volcano map for CREB5 in TCGA database. **B** Heat map of differentially expressed genes after CREB5 overexpression in HepG2 cells. **C** Venn diagram of intersection of different gene sets. The effect of CREB5 knockdown (**D**) or overexpression (**E**) on TNC protein level was detected by western-blotting. **F** Chord diagram of GO enrichment analysis. GSEA map showing a significant correlation between TNC expression level and extracellular matrix organization (**G**) and focal adhesion PI3K-AKT-mTOR signaling pathway (**H**) (TCGA). **I** Schematic diagram of dual-luciferase reporter assay. **J** Co-transfection with CREB5 expression vector increased TNC promoter activity in 293T cells compared with that by blank vector. **K** ChIP-qPCR revealed the potential binding sites of CREB5 in the TNC promoter region.

To explore the downstream target genes of CREB5, we first analyzed the gene expression profiles of the CREB5 high-expression and low-expression groups in TCGA and detected 1095 differential genes, including 961 upregulated genes (Log_2_FC ≥ 1, p.adj < 0.05) (Fig. 7A). Second, we analyzed the differences in gene expression profiles between CREB5-overexpressing and control cells using RNA-seq, which identified 2724 DEGs (687 upregulated genes and 2037 downregulated genes) (|Log_2_FC| ≥ 1, p.adj < 0.05) (Fig. 7B, Supplementary Fig. 4A). KEGG enrichment analysis showed that the DEGs were mainly enriched in the PI3K-AKT signaling pathway, ECM receptor interaction, focal adhesion, and cancer pathway (Supplementary Fig. 4B). Simultaneously, we obtained an EMT core gene set from the EMT-related database, dbEMT, and found that the upregulated genes identified in TCGA and RNA-seq analysis had some overlap with the EMT-related gene set, namely TNC, TBX20, and PDGFD (Fig. 7C). To further confirm the target genes of CREB5, we knocked down or overexpressed CREB5 in several HCC cell lines. RT-qPCR analysis showed that only TNC decreased with CREB5 knockdown and increased with CREB5 overexpression, while neither TBX20 nor PDGFD were fully satisfied (Supplementary Fig. 4C, D). We further verified that TNC protein levels varied with changes in CREB5 protein levels by western blot (Fig. 7D, E). Similar results were observed in the mouse liver cancer cell line Hep1-6 (Supplementary Fig. 4E, F). We also constructed a GO chord diagram based on the enriched pathways of CREB5 and found that TNC was a common gene in multiple pathways (Fig. 7F). Therefore, we considered TNC as a promising potential effector of CREB5. Most interestingly, GSEA enrichment showed that TNC was closely related to ECM organization, focal adhesion, and the PI3K-AKT-mTOR signaling pathway (Fig. 7G, H). Correlation analysis based on TCGA data showed that CREB5 was positively correlated with TNC (*R* = 0.448, *p* < 0.001) (Supplementary Fig. 4G). Additionally, ERS increased TNC expression, and this upregulation was inhibited by 4-PBA (Supplementary Fig. 4H, I).

Given that CREB5 is a transcription factor that regulates TNC in liver cancer cells, we performed a dual-luciferase reporter assay to investigate whether CREB5 increases TNC promoter activity. The full-length TNC promoter fragment was subcloned into a luciferase reporter (Fig. 7I). Compared to the blank vector, co-transfection of 293T cells with a CREB5 expression vector increased TNC promoter activity (Fig. 7J). We used the JASPAR database (http://jaspar.genereg.net/) to search the TNC promoter region for CREB5 binding sites, and identified three putative binding sites (sites 1–3) and thus designed and synthesized corresponding primers. The results of ChIP-qPCR showed that CREB5 had the highest enrichment at site 2 (Fig. 7K).

### CREB5 promotes EMT of liver cancer cells through TNC

**Fig. 8:**
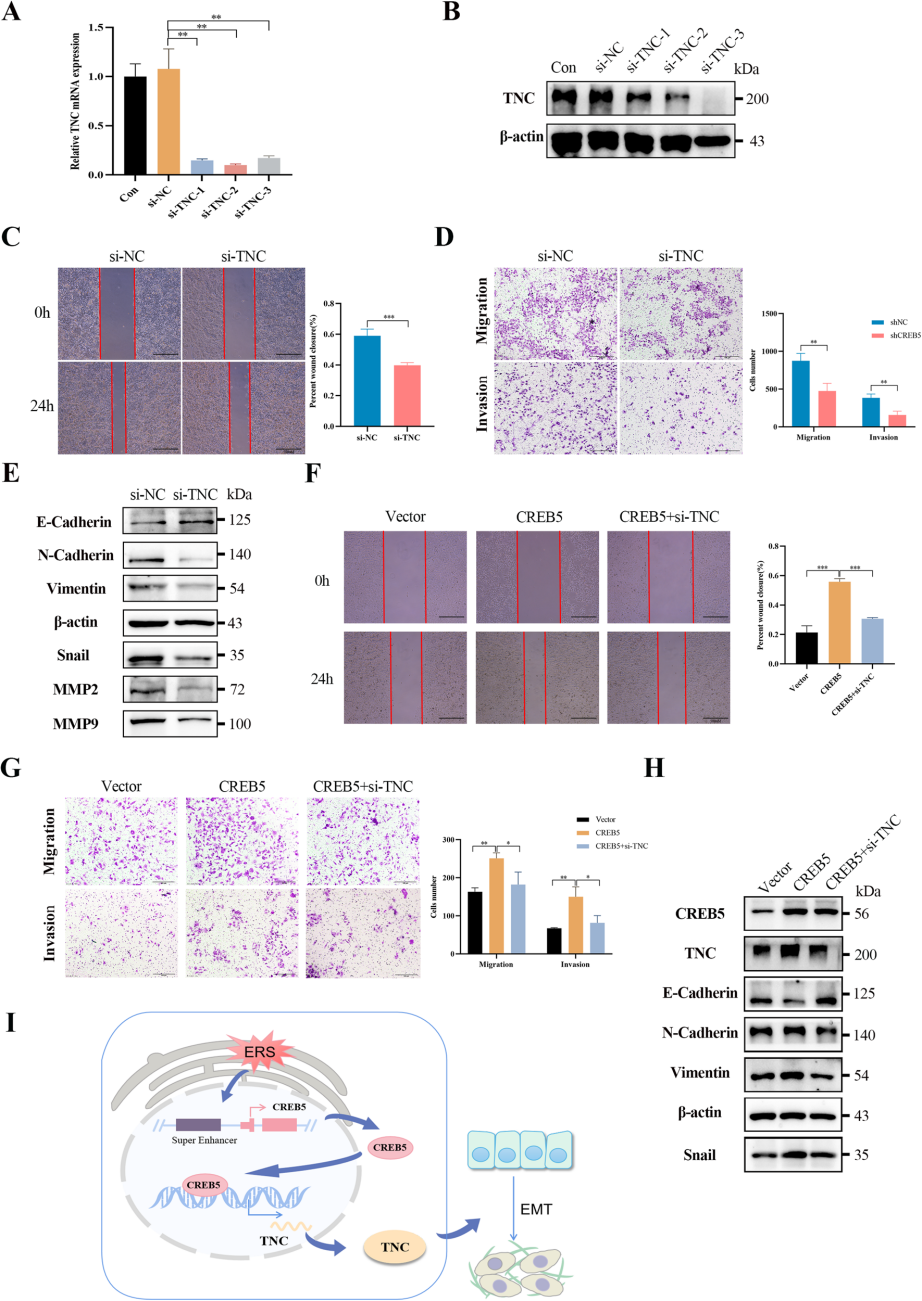
CREB5 promotes EMT of liver cancer cells through TNC. Detection of TNC mRNA (**A**) and protein (**B**) expression after RNA interference in Hep3B cells. **C** The migration ability of Hep3B cells after TNC interference was detected by wound-healing assay. **D** The migration and invasion ability of Hep3B after TNC interference was detected by transwell assay. **E** Relative protein expression levels of EMT markers after TNC interference. The TNC gene was knocked down in cell lines with stable overexpression of CREB5, followed by wound-healing assay (**F**), transwell assay (**G**), and detection of EMT-related marker proteins (**H**). **I** Proposed working model of transcriptional regulation in HCC biology.

To further explore the role of TNC in promoting EMT in HCC, we used siRNA to knockdown TNC in Hep3B cells and verified the knockdown efficiency using RT-qPCR and western blotting (Fig. 8A, B). Further results showed that knockdown of TNC significantly inhibited cell migration and invasion (Fig. 8C, D), and reduced the protein levels of EMT, MMP2, and MMP9 (Fig. 8E). In addition, flow cytometry showed that TNC knockdown promoted apoptosis of Hep3B cells and increased the sensitivity of Hep3B cells to lenvatinib (Supplementary Fig. 4J, K). We then knocked down TNC expression in HepG2 cells stably overexpressing CREB5. Compared to that in control cells, CREB5 overexpression significantly promoted invasion and migration, and this effect was inhibited by knockdown of TNC (Fig. 8F, G). The CREB5-mediated promotion of EMT was inhibited to varying degrees following knockdown of TNC (Fig. 8H).

## Discussion

Several studies have highlighted the role of ERS in tumor progression, particularly its role in facilitating tumor cell migration and invasion, which are essential for metastasis. SEs are large clusters of transcriptional enhancers that drive the expression of crucial genes involved in cell identity, survival, and metastasis, particularly in cancer. However, whether specific SE are involved in the progression of EMT associated with ERS has not been reported. Here, we provide the first evidence that activation of ERS promotes EMT in HCC cells via a key target gene of ERS-specific SE, CREB5, which is significantly upregulated in HCC tissues and is associated with poor prognosis and an aggressive phenotype. CREB5 binds directly to the promoter region of TNC and promotes its transcription, thereby promoting EMT (Fig. 8I). Our results provide new information on the mechanism by which ERS regulates EMT and valuable insights for developing strategies to block tumor metastasis.

It is important to understand the pathogenic mechanisms and epigenetic changes that drive HCC recurrence and metastasis. EMT plays a key role in tumor invasion and metastasis and contributes to the progression of HCC [27]. Recent studies have shown that SEs can promote EMT by driving the transcription of EMT-related transcription factors [28, 29]. In non-small cell lung cancer, SE promote TGFβ-induced EMT by enhancing the expression of EMT-related transcription factors, while a BRD4-specific inhibitor JQ1 can inhibit SE-driven transcription of ETS2, HNF4A, JUNB, and other genes, resulting in the obstruction of EMT process [30]. In HCC, SE reportedly activate the Akt/GSK-3β/Snail signaling pathway by regulating the transcription of the target gene, AJUBA, thereby inducing EMT and ultimately promoting the invasion and metastasis of HCC cells [16]. Consistent with the previous results, in the present study, we found that SE is regulated by ERS and induces EMT in HCC cells by affecting the transcription of the key target gene CREB5, which has been indicated as a stem cell-like factor in gliomas and prostate cancer [18, 31]. Further, we found that CREB5, as a transcription factor, was highly expressed in HCC and played a role in promoting cell proliferation, migration, invasion and inhibiting cell apoptosis in different HCC cell lines. Overexpression of CREB5 promoted EMT in HCC cells. These results suggest that ERS is highly correlated with EMT and invasiveness in liver cancer by targeting SE and the target gene CREB5.

TNC is an ECM glycoprotein and abnormally high expression of TNC has been associated with numerous diseases, including several cancers. For instance, TNC is highly expressed in breast [32], esophageal [33], liver [34], colorectal [35], and gastric cancers [36] and is associated with malignant progression and poor prognosis. TNC binds directly to αvβ1, αvβ6, and annexin II [37] and activates the JNK/c-Jun signaling pathway [38] and SRC expression, which further resulting in EMT [39]. Here, a single gene difference analysis of CREB5 using TCGA datasets, combined with RNA-seq data and EMT-related gene set analysis, revealed TNC as a key target gene of CREB5 in the regulation of EMT. Our study also confirmed that TNC mRNA and protein levels were regulated by CREB5. Moreover, dual-luciferase and ChIP-qPCR assays verified that CREB5 binds to the TNC promoter, promotes transcription of TNC, and plays a role in inducing EMT in liver cancer cells. Knockdown of TNC inhibited CREB5-induced promotion of liver cancer cell migration, invasion, and EMT, which further indicated that CREB5 plays a role in promoting EMT through TNC. In addition, TNC knockdown promoted apoptosis and increased the sensitivity of Hep3B cells to lenvatinib.

Although this study identified that CREB5, targeted by ERS-related SE, promotes EMT in liver cancer cells by directly activating the transcription of TNC, several questions remain. First, the complex relationship between ERS and EMT needs to be studied further, as multiple mechanisms may be activated simultaneously rather than being associated with the regulation of a single pathway. In addition, since SE usually regulates the expression of related genes in 3D structure, how SE interacts with the transcription start site of CREB5 in 3D space to regulate its transcription needs further investigation. Nevertheless, the above issues still need to be further explored, our study provides new insights into the mechanism by which ERS promotes EMT and highlights super-enhancers and CREB5 as potential therapeutic targets for HCC.

## Materials and methods

### Study subjects, samples, and datasets

Microarray analysis of human liver cancer and adjacent normal liver tissues was performed at the First Affiliated Hospital of Anhui Medical University. The experimental protocol was approved by the Ethics Committee of Anhui Medical University (NO.20040158), and clinical specimens were collected after obtaining written informed consent from the patients. The clinical data collected included age, sex, history of hepatitis and cirrhosis, degree of differentiation, tumor size, and clinical stage. We also obtained eight pairs of fresh primary liver cancer and adjacent normal liver tissue samples from the First Affiliated Hospital of Anhui Medical University.

RNA-seq data and clinical information were obtained from The Cancer Genome Atlas (TCGA) database (https://portal.gdc.com). EMT data were obtained from the EMT-related database, dbEMT (http://dbemt.bioinfo-minzhao.org/index.html) [40, 41].

### Cell culture

HepG2, Hep3B, Huh7, LM3, Hep1-6, and MHCC-97H cell lines (Chinese Academy of Sciences, Shanghai, China) were tested for mycoplasma negativity. These cells were cultured in high-glucose DMEM (Wisent, Canada) containing 1% streptomycin (Beyotime, Shanghai, China), 1% penicillin (Beyotime), and 10% fetal bovine serum (Wisent). Cells were cultured at 37 °C with 5% CO_2_. The medium was replaced as needed according to cell growth.

### Western blotting

Total protein was extracted using RIPA buffer containing 1% PMSF (Beyotime, Shanghai, China). After lysis on ice for 30 min and centrifugation at 14,000 rpm for 15 min, the protein-containing supernatant was collected. An aliquot of the extract was mixed with 5× SDS-PAGE sample loading buffer and heated at 100 °C for 10 min. Proteins were separated by SDS-PAGE and transferred to PVDF membranes. After blocking for 1 h, the blot was incubated with a specific primary antibody at 4 °C overnight. After washing three times with TBST, the blot was incubated with the corresponding secondary antibody at room temperature for 2 h. Enhanced chemiluminescence detection reagent (Thermo Fisher, MA, USA) was used to visualize the target protein bands. The antibodies used in this study and their dilution ratios are listed in Supplementary Table 3.

### RT-qPCR

Total RNA was extracted using TRIzol reagent (Invitrogen, California, USA) and used to synthesize complementary DNA (cDNA) using ToloScript All-in-one RT EasyMix for qPCR (TOLOBIO, Shanghai, China). RT-qPCR was performed using 2× Q3 SYBR qPCR Master Mix (TOLOBIO, Shanghai, China) according to the manufacturer’s instructions. Primer sequences are listed in Supplementary Table 4.

### dCas9-KRAB

SEs were edited using CRISPR/Cas9 repressor. Three sgRNAs (listed in Supplementary Table 6) targeting the CREB5 SE region were cloned into the Lenti-CAS9-puro plasmid and co-transfected with the packaging plasmid into 293T cells to generate a virus expressing Cas9 and the sgRNAs. HepG2 cells were infected with the generated viruses, and G418 was used for selection to produce homozygous clonal cells with specific silenced SE regions.

### Immunohistochemical (IHC) staining

The expression levels of CREB5 and GRP78 in paraffin-embedded tissue microarrays of HCC and adjacent non-tumor liver tissues were detected using immunohistochemical staining (Universal Two-step Detection Kit; ZSGB-GIO, Beijing, China) according to the manufacturer’s instructions. Cell staining was scored according to intensity (negative, 0; weak, 1; moderate, 2; or strong, 3) and the percentage of positive cells (<10%, 0; 10%–25%, 1; 26%–50%, 2; 51%–75%, 3; or >75%, 4). The final IHC score was the product of these two measures. We defined ≥6 as high expression and <6 as low expression. Investigator were blinded to the group allocation when scoring.

### Small interfering RNA (SiRNA) and plasmid transfection

CREB5, TNC, and negative control (NC) siRNAs were purchased from GenePharma (Shanghai, China), and a CREB5 overexpression plasmid and corresponding GV658 empty vector were purchased from Genechem (Shanghai, China). siRNA target sequences are listed in Supplementary Table 5.

### Lentivirus transfection

Lentiviruses encoding CREB5 short hairpin RNA (shRNA) and overexpression constructs were designed and synthesized by Gemma Corporation (Shanghai, China). The sequences of the shRNA targets are shown in Supplementary Table 6. The effectiveness of lentiviral infection was verified by RT-qPCR and western blotting.

### Cell viability analysis

Stably transfected cells (100 µl) were seeded into a 96-well plate. Cell Counting Kit-8 reagent (NCM Biotech, Suzhou, China) was used for detection according to the instructions of the manufacturer. After incubation, the absorbance at 450 nm was measured.

### Colony formation assay

Stably transfected cells (500–1000 cells) were seeded in 6-well plates and incubated for 10–14 days. The medium was changed according to their growth state until macroscopic colony formation was observed. After fixation and staining, the cell colony formation was observed.

### 5-Ethynyl-2′-deoxyuridine (EdU) assay

Stably transfected cells were seeded in 24-well plates lined with glass plates. After 24 h, EdU reagent was added, and the cells were incubated for 2 h. Then, cell proliferation was evaluated using the BeyoClick EdU Cell Proliferation Kit with Alexa Fluor 555 (Beyotime) according to the manufacturer’s protocol. The specimens were photographed, counted, and analyzed using a fluorescence microscope.

### Wound healing assay

When the confluence rate of stably transfected cells in 6-well plates reached 90%, a wound was created by scratching using a 200 µl pipette tip. At 0 and 24 h of incubation, pictures were taken under a microscope. ImageJ software was used for analysis.

### Migration and invasion assays

Migration assays were performed using 8 μm transwell chambers (Corning, USA) coated with Matrigel (Corning) to assess cell invasion. Cells were seeded in the upper chamber and incubated at 37 °C for 24 h. Migrated cells were fixed, stained with 0.1% crystal violet, photographed, and counted under a microscope.

### Annexin V-FITC/PI apoptosis detection

After treatment, the cells were collected in centrifuge tubes, and single-cell suspensions were prepared. Apoptosis was detected by Annexin V-FITC apoptosis detection kit (BB-4101-2, BestBio, Shanghai, China). Data were obtained using a CytoFLEXLX flow cytometer (Beckman Coulter) and analyzed using CytExpert software.

### Immunofluorescence

Cells were fixed with 4% paraformaldehyde for 20 min at room temperature, permeabilized with 0.5% Triton X-100 phosphate-buffered saline (PBS) for 15 min at room temperature, and blocked with goat serum for 30 min. After overnight incubation with an anti-CREB5 primary antibody at 4 °C and three washes with PBS, the cells were stained with Alexa Fluor 488 goat-conjugated anti-rabbit IgG secondary antibody for 2 h at room temperature. Finally, nuclei were stained with DAPI (Beyotime) for 8 min, and cells in random fields were photographed under an Olympus confocal laser scanning microscope.

### Dual-luciferase reporter assay

The TNC promoter sequence (−2000 to +30) was synthesized, digested, and cloned into a GV534 plasmid (GeneChem, Shanghai, China). The primer sequences are listed in Supplementary Table 7. Luciferase reporter plasmids were transiently transfected into 293T cells using Lipofectamine 2000 (Invitrogen). At 48 h after transfection, luciferase activity was measured using the Dual-Glo Luciferase Assay system (Promega, Madison, WI, USA) according to the manufacturer’s guidelines.

### ChIP-qPCR

For ChIP-qPCR, immunoprecipitation was performed overnight at 4 °C using anti-CREB5 or anti-IgG antibodies. Then, immunoprecipitated DNA was quantified using RT-qPCR, and the data were normalized to the input. The primers used for ChIP-qPCR are listed in Supplementary Table 7.

### Animal experiments

Animal experiments were approved by the Animal Ethics Committee of Anhui Medical University (NO.20242202). BALB/c nude mice (4–5 weeks old, *n* = 20) were purchased from GemPharmatech Co. Ltd (Nanjing, China). Twenty mice were randomly divided into four groups. Hep3B cells (3 × 10^6^ cells) transfected with the control (shNC) or the CREB5 stable-knockdown shRNA (shCREB5) were resuspended in 100 µl of PBS and subcutaneously injected into the right axilla of the mice. HepG2 cells (3 × 10^6^ cells) stably overexpressing CREB5 (CREB5) or control cells were resuspended in 100 µl of PBS and subcutaneously injected into the right axilla of the mice. After 20 days, when the tumor had grown to 1 cm^3^ (L × W^2^/2), the mice were euthanized, and tumor tissues were removed, weighed, and recorded. Investigators were blinded to the group allocation when assessing the results. The tumor tissues were embedded in paraffin, sectioned, and subjected to IHC staining.

### Statistical analyses

The experimental data are shown as the mean ± SD. A *t*-test was used to compare the means of two samples, and ANOVA was used to compare the means of multiple groups. Chi-square tests were used to determine the correlations between protein expression and clinical parameters. Overall survival (OS) was analyzed using the Kaplan–Meier method. GraphPad Prism software (version 10.0) was used for data plotting and statistical analyses. Each experiment was repeated thrice. A *p* value < 0.05 was considered statistically significant. Statistical significance is indicated as follows: *p < 0.05, **p < 0.01, and ***p < 0.001.

## Supplementary information

Supplementary figure legends

Supplementary Figure 1

Supplementary Figure 2

Supplementary Figure 3

Supplementary Figure 4

Supplementary Table 1

Supplementary Table 2

Supplementary Table 3

Supplementary Table 4

Supplementary Table 5

Supplementary Table 6

Supplementary Table 7

Original western blots

## Data Availability

All data from the current study and/or the analysis period are included in this publication and supplementary information file. Data sets for the study are available upon reasonable request. The GEO accession number for mRNA-sequencing data of CREB5 overexpression was GSE280778. The GEO accession numbers of RNA-seq and ChIP-seq data for TM-treated and untreated HepG2 cells were GSE208391 and GSE209520, respectively.
