# Supplementary material for: Endoplasmic reticulum stress-related super enhancer promotes epithelial-mesenchymal transformation in hepatocellular carcinoma through CREB5 mediated activation of TNC
Source: Cell Death Dis. 2025 Feb 6;16(1):73. doi: 10.1038/s41419-025-07356-y (PMC11802765; doi:10.1038/s41419-025-07356-y)
Supplement: Supplementary file 1 — Supplementary figure legends [file 41419_2025_7356_MOESM1_ESM.docx]

Supplemental Figure 1. **A** Expression of 17 genes in HCC and normal liver tissue (TCGA). **B** GO, KEGG enrichment analysis was performed on 17 ERS-specific SEs target genes. **C** Relative mRNA expression of BRD4 and EMT markers (E-cadherin, N-cadherin, and vimentin) in HepG2 cells treated with JQ1. **D** Kaplan-Meier survival curves of disease specific survival based on TCGA database. **E** Kaplan-Meier survival curves of progress free interval based on TCGA database. **F** LM3 cells were treated with TM (2.5 µM) or TM (2.5 µM)+4-PBA (1 mM), and the expression of CREB5 was detected by immunofluorescence. **G** Protein and mRNA expression of CREB5 in five liver cancer cell lines.

Supplemental Figure 2. **A, B** Protein (A) and mRNA (B) levels were detected after CREB5 knockdown in Hep3B and LM3 cells. **C-E** The effect of CREB5 knockdown on the proliferation of LM3 cells was detected by CCK-8 assay (C), colony formation assay (D) and EDU (E). **F, G** Detection of protein (F) and mRNA (G) levels after overexpression of CREB5 in HepG2 and Huh7 cells. **H-J** The effect of CREB5 overexpression on the proliferation of Huh7 cells was detected by CCK-8 assay (H), colony formation assay (I) and EDU (J).

Supplemental Figure 3. **A-D** GSEA analysis indicated significant correlations between CREB5 and ECM receptor interaction (A), extracellular matrix organization (B), matrix metalloproteinases (C), PI3K-AKT signaling pathway (D). **E, F** The mRNA (E) and protein (F) levels after CREB5 knockdown or overexpression were detected in Hep1-6 cells. **G, H** Relative mRNA (G) and protein (H) expression levels of EMT markers after knockdown or overexpression of CREB5 in Hep1-6 cells. **I, J** Relative mRNA (I) and protein (J) expression levels of MMP2, MMP9 after knockdown or overexpression of CREB5 in Hep1-6 cells. (EV: empty vector)

Supplemental Figure 4. **A** Volcano plot of differentially expressed genes in HepG2 cells after CREB5 overexpression. **B** KEGG enrichment analysis showed CREB5-related signaling pathways. **C, D** The mRNA levels of TNC, TBX20, and PDGFD were evaluated in Hep3B and LM3 cell lines with CREB5 knockdown (C), as well as in HepG2 and huh7 cell lines with CREB5 overexpression (D). **E** Effects of CREB5 knockdown on TNC mRNA and protein levels in Hep1-6 cells. **F** Effects of CREB5 overexpression on TNC mRNA and protein levels in Hep1-6 cells. **G** The correlation between CREB5 and TNC in HCC according to mRNA expression in TCGA database. **H, I** Changes in GRP78 and the TNC mRNA (H) and protein (I) level after the HepG2 cells were treated with TM (2.5 µM) or TM (2.5 µM) + 4-PBA (1 mM). **J, K** After interfering TNC expression in Hep3B cells, the sensitivity of lenvatinib was detected by flow cytometry. (EV: empty vector)
