# Supplementary figures and images for: Endoplasmic reticulum stress-related super enhancer promotes epithelial-mesenchymal transformation in hepatocellular carcinoma through CREB5 mediated activation of TNC
Source: Cell Death Dis. 2025 Feb 6;16(1):73. doi: 10.1038/s41419-025-07356-y (PMC11802765; doi:10.1038/s41419-025-07356-y)

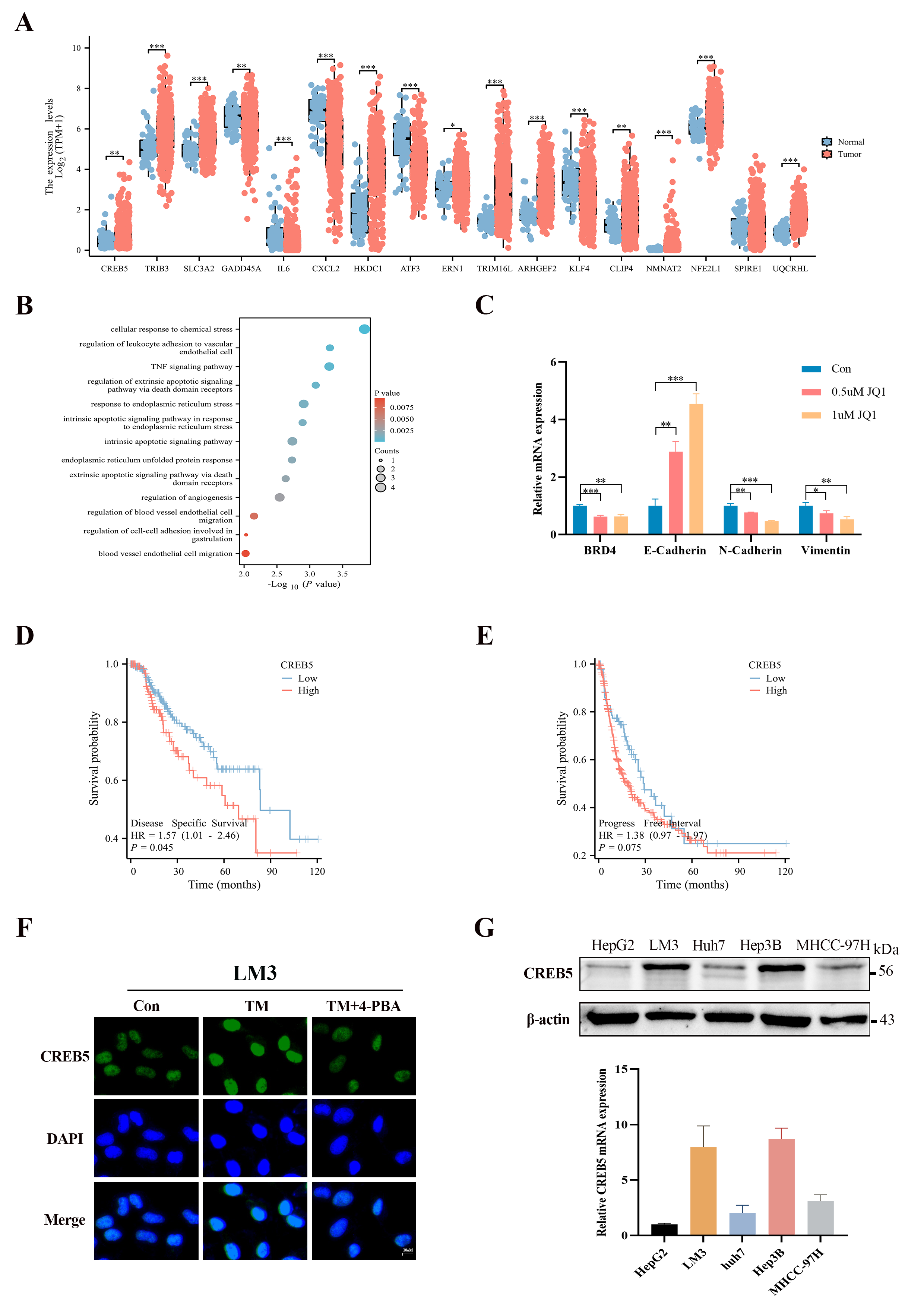

Supplement: Supplementary file 2 — Supplementary Figure 1 [file 41419_2025_7356_MOESM2_ESM.tif]

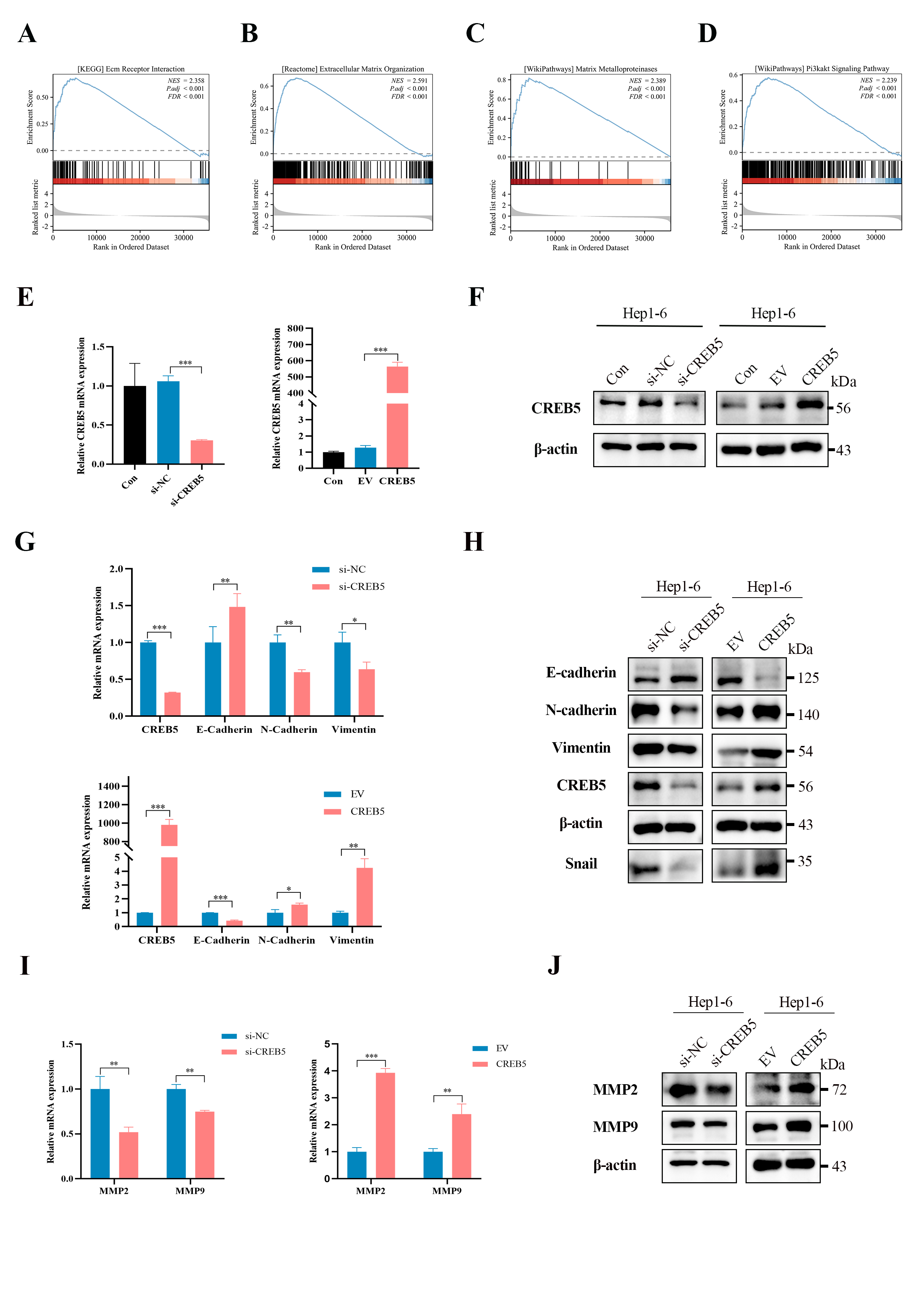

Supplement: Supplementary file 4 — Supplementary Figure 3 [file 41419_2025_7356_MOESM4_ESM.tif]

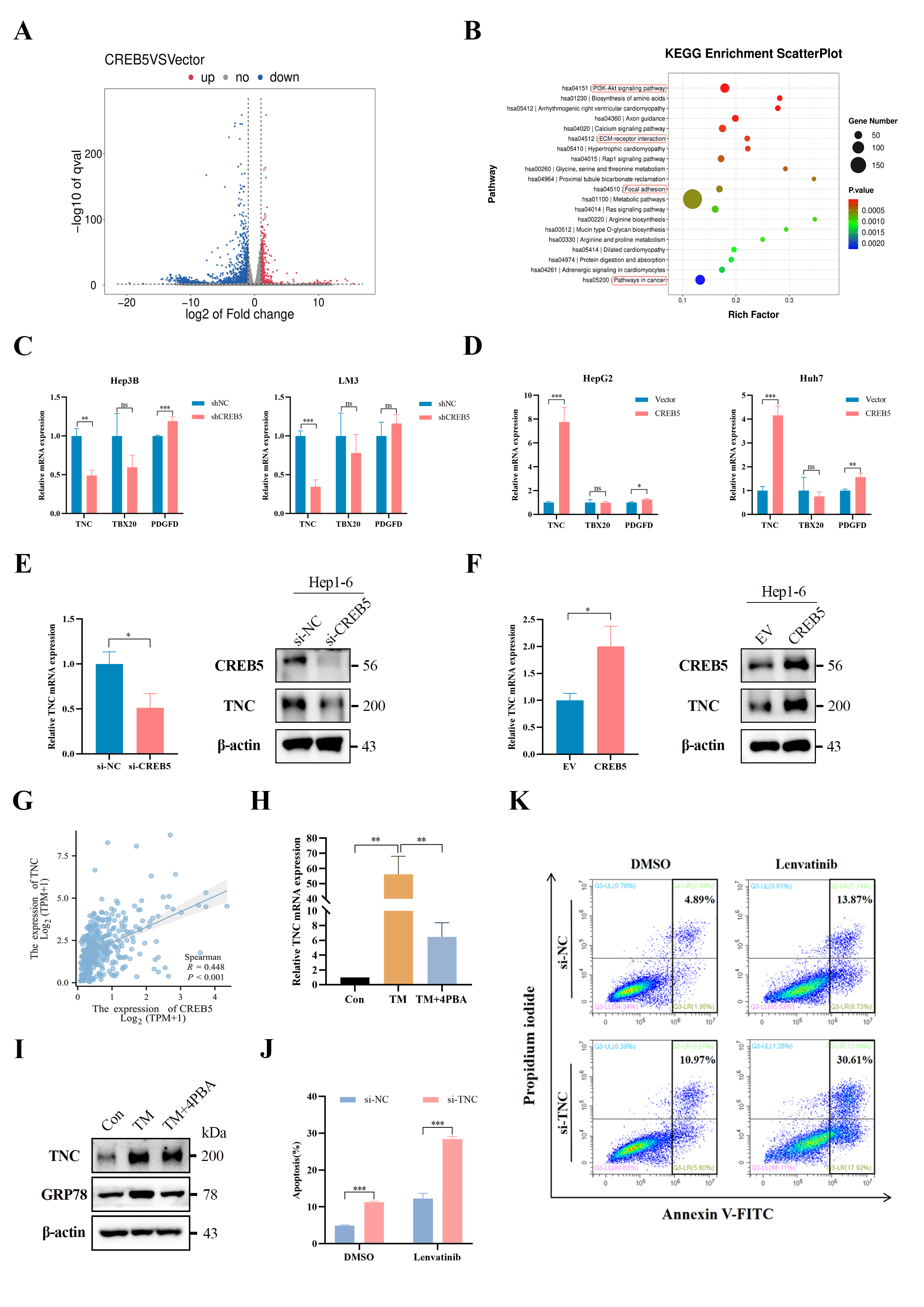

Supplement: Supplementary file 5 — Supplementary Figure 4 [file 41419_2025_7356_MOESM5_ESM.tif]
