# Supplementary material for: Endoplasmic reticulum stress-related super enhancer promotes epithelial-mesenchymal transformation in hepatocellular carcinoma through CREB5 mediated activation of TNC
Source: Cell Death Dis. 2025 Feb 6;16(1):73. doi: 10.1038/s41419-025-07356-y (PMC11802765; doi:10.1038/s41419-025-07356-y)
Supplement: Supplementary file 6 — Supplementary Table 1 [file 41419_2025_7356_MOESM6_ESM.docx]

**Table S1. Correlation between CREB5 expression and clinicopathological features of HCC patients.**

| Variable | No. of patients | CREB5 expression | | p |
| --- | --- | --- | --- | --- |
|  |  | High(n,%) | Low(n,%) |  |
| **Age(years)** | | | | |
| <60 | 64 | 39,60.94% | 25,39.06% | 0.8821 |
| ≥60 | 32 | 20,62.50% | 12,37.50% |  |
| **Gender** | | | | |
| Male | 72 | 48,66.67% | 24,33.33% | 0.0694 |
| Female | 24 | 11,45.83% | 13,54.17% |  |
| **History of hepatitis** | | | | |
| Yes | 71 | 46,64.79% | 25,35.21% | 0.2585 |
| No | 25 | 13,52.00% | 12,48.00% |  |
| **History of cirrhosis** | | | | |
| Yes | 45 | 29,64.44% | 16,35.56% | 0.5723 |
| No | 51 | 30,58.82% | 21,41.18% |  |
| **Tumor size(cm)** | | | | |
| <5 | 33 | 15,45.45% | 18,54.55% | 0.0250 |
| 5~10 | 45 | 29,64.44% | 16,35.56% |  |
| ≥10 | 18 | 15,83.33% | 3,16.67% |  |
| **Degree of differentiation** | | | | |
| High | 32 | 11,34.38% | 21,65.62% | 0.0004 |
| Moderate | 50 | 36,72.00% | 14,28.00% |  |
| Low | 14 | 12,85.71% | 2,14.29% |  |
| **AFP level** | | | | |
| High | 35 | 22,62.86% | 13,37.14% | 0.2334 |
| Moderate | 25 | 12,48.00% | 13,52.00% |  |
| Low | 36 | 25,69.44% | 11,30.56% |  |
