# Supplementary material for: Endoplasmic reticulum stress-related super enhancer promotes epithelial-mesenchymal transformation in hepatocellular carcinoma through CREB5 mediated activation of TNC
Source: Cell Death Dis. 2025 Feb 6;16(1):73. doi: 10.1038/s41419-025-07356-y (PMC11802765; doi:10.1038/s41419-025-07356-y)
Supplement: Supplementary file 7 — Supplementary Table 2 [file 41419_2025_7356_MOESM7_ESM.docx]

**Table S2. Gene co-occurrent alteration between CREB5 and GRP78**

| CREB5 | GRP78 | | χ^2^ | P value |
| --- | --- | --- | --- | --- |
|  | Positive | Negative |  |  |
| Positive | 47 | 12 | 15.21 | <0.001 |
| Negative | 15 | 22 |  |  |
