# Supplementary material for: Endoplasmic reticulum stress-related super enhancer promotes epithelial-mesenchymal transformation in hepatocellular carcinoma through CREB5 mediated activation of TNC
Source: Cell Death Dis. 2025 Feb 6;16(1):73. doi: 10.1038/s41419-025-07356-y (PMC11802765; doi:10.1038/s41419-025-07356-y)
Supplement: Supplementary file 8 — Supplementary Table 3 [file 41419_2025_7356_MOESM8_ESM.docx]

**Table S3. Information of antibodies**

| Antibodies | Source | Catalogue # | Concentration | | |
| --- | --- | --- | --- | --- | --- |
|  |  |  | Western blot | ICC | IHC |
| Rabbit polyclonal, CREB5 | Bioss | bs-14053R | N/A | 1:200 | 1:200 |
| Rabbit polyclonal, CREB5 | Proteintech | 14196-1-AP | 1:1000 | N/A | N/A |
| Mouse monoclonal, CRE-BPa | Santa Cruz Biotechnology | sc-130435 | ChIP-qPCR | | |
| Rabbit monoclonal,BRD4 | Abcam | ab128874 | 1:2000 | N/A | N/A |
| Rabbit polyclonal,GRP78 | Abcam | ab21685 | 1:1000 | N/A | 1:200 |
| Rabbit polyclonal, E-cadherin | Proteintech | 20874-1-AP | 1:10000 | N/A | N/A |
| Rabbit polyclonal, N-cadherin | ABclonal | A19083 | 1:2000 | N/A | N/A |
| Rabbit polyclonal, Vimentin | Abmart | T55134F | 1:5000 | N/A | N/A |
| Rabbit polyclonal,Snail | ABclonal | A11794 | 1:1000 | N/A | N/A |
| Rabbit polyclonal,MMP9 | ABclonal | A2095 | 1:1000 | N/A | N/A |
| Rabbit polyclonal,MMP2 | ABclonal | A6247 | 1:2000 | N/A | N/A |
| Rabbit monoclonal,Tenascin C | Cell Signaling Technology | 33352S | 1:1000 | N/A | N/A |
| Rabbit monoclonal,Bax | Cell Signaling Technology | 5023 | 1:1000 | N/A | N/A |
| Rabbit polyclonal,Bcl-2 | Affinity | AF6139 | 1:1000 | N/A | N/A |
| Mouse monoclonal,Ki-67 | Cell Signaling Technology | 9949 | N/A | N/A | 1:500 |
| Anti-rabbit IgG (H+L), F(ab')2 Fragment (Alexa Fluor® 488 Conjugate) | Cell Signaling Technology | 4412 | N/A | 1:1000 | N/A |
| Goat anti-Mouse secondary antibodies/TRITC | ZSGB-BIO | ZF-0316 | 1:100000 | N/A | N/A |
| Goat anti-Rabbit secondary antibodies/TRITC | ZSGB-BIO | ZF-0313 | 1:100000 | N/A | N/A |
| Mouse monoclonal, β-actin | ZSGB-BIO | TA-09 | 1:2000 | N/A | N/A |
