# Supplementary material for: Endoplasmic reticulum stress-related super enhancer promotes epithelial-mesenchymal transformation in hepatocellular carcinoma through CREB5 mediated activation of TNC
Source: Cell Death Dis. 2025 Feb 6;16(1):73. doi: 10.1038/s41419-025-07356-y (PMC11802765; doi:10.1038/s41419-025-07356-y)
Supplement: Supplementary file 9 — Supplementary Table 4 [file 41419_2025_7356_MOESM9_ESM.docx]

**Table S4. Primer sequences for RT-qPCR**

| Target gene | Forward (5’-3’) | Reverse (5’-3’) |
| --- | --- | --- |
| CREB5 | CCCTGCCCAACCCTACAATG | GGACCTTGCATCCCCATGAT |
| β-actin | CCCAGCCATGTACGTTGCTA | TCACCGGAGTCCATCACGAT |
| E-cadherin | CGAGAGCTACACGTTCACGG | GGGTGTCGAGGGAAAAATAGG |
| N-cadherin | TTTGATGGAGGTCTCCTAACACC | ACGTTTAACACGTTGGAAATGTG |
| Vimentin | GAGAACTTTGCCGTTGAAGC | GCTTCCTGTAGGTGGCAATC |
| MMP2 | CCCACTGCGGTTTTCTCGAAT | CAAAGGGGTATCCATCGCCAT |
| MMP9 | TGTACCGCTATGGTTACACTCG | GGCAGGGACAGTTGCTTCT |
| TBX20 | TCGTCCCTGTGGACAACAAG | TTCAGGTTGAGCAATGAGGCT |
| PDGFD | ACGGATACAGCTAGTGTTTGACA | GTCCACACCATCGTCCTCTAATA |
| BRD4 | GGCCTGTGAAACCTCCAAAG | TCCACAGGCTTGTAGAAGGG |
| TNC | TCCCAGTGTTCGGTGGATCT | TTGATGCGATGTGTGAAGACA |
| CREB5 (mouse) | GTCCCAGGCTCTCTATCATCTC | ATAGGCATCAAGACGGCAGAA |
| β-actin (mouse) | CTACCTCATGAAGATCCTGACC | CACAGCTTCTCTTTGATGTCAC |
| E-cadherin(mouse) | CAGTTCCGAGGTCTACACCTT | TGAATCGGGAGTCTTCCGAAAA |
| N-cadherin (mouse) | AGGCTTCTGGTGAAATTGCAT | GTCCACCTTGAAATCTGCTGG |
| Vimentin (mouse) | CGTCCACACGCACCTACAG | GGGGGATGAGGAATAGAGGCT |
| MMP2 (mouse) | ACCTGAACACTTTCTATGGCTG | CTTCCGCATGGTCTCGATG |
| MMP9 (mouse) | CTGGACAGCCAGACACTAAAG | CTCGCGGCAAGTCTTCAGAG |
| TNC (mouse) | TTTGCCCTCACTCCCGAAG | AGGGTCATGTTTAGCCCACTC |
