# Supplementary material for: Endoplasmic reticulum stress-related super enhancer promotes epithelial-mesenchymal transformation in hepatocellular carcinoma through CREB5 mediated activation of TNC
Source: Cell Death Dis. 2025 Feb 6;16(1):73. doi: 10.1038/s41419-025-07356-y (PMC11802765; doi:10.1038/s41419-025-07356-y)
Supplement: Supplementary file 10 — Supplementary Table 5 [file 41419_2025_7356_MOESM10_ESM.docx]

**Table S5. Sequences of siRNA**

| Gene | Sequences | |
| --- | --- | --- |
|  | Sense (5’-3’) | Antisense (5’-3’) |
| Negative control | UUCUCCGAACGUGUCACGUTT | ACGUGACACGUUCGGAGAATT |
| CREB5-1 | GAUGCAUAAUGCAGUUGGUTT | ACCAACUGCAUUAUGCAUCTT |
| CREB5-2 | CAGCAACACAACAGAUGCATT | UGCAUCUGUUGUGUUGCUGTT |
| CREB5-3 | CAGCAUAAUACCAUCACUATT | UAGUGAUGGUAUUAUGCUGTT |
| TNC-1 | GGAGUACUUUAUCCGUGUA | UACACGGAUAAAGUACUCC |
| TNC-2 | GACGAUGCGUGGAGAAUGA | UCAUUCUCCACGCAUCGUC |
| TNC-3 | GGGCUAUAGAACACCAGUA | UACUGGUGUUCUAUAGCCC |
