# Supplementary material for: Endoplasmic reticulum stress-related super enhancer promotes epithelial-mesenchymal transformation in hepatocellular carcinoma through CREB5 mediated activation of TNC
Source: Cell Death Dis. 2025 Feb 6;16(1):73. doi: 10.1038/s41419-025-07356-y (PMC11802765; doi:10.1038/s41419-025-07356-y)
Supplement: Supplementary file 11 — Supplementary Table 6 [file 41419_2025_7356_MOESM11_ESM.docx]

**Table S6. Sequences of shRNAs and sgRNAs**

| Target gene | Sequence(5’-3’) |
| --- | --- |
| shNC | TTCTCCGAACGTGTCACGT |
| shCREB5 | CAGCATAATACCATCACTA |
| CREB5-Enhancer-sgRNA-1 | ACAGCTGTTTAATGACACTCT |
| CREB5-Enhancer-sgRNA-2 | AAACAAGAGCTGAACCTATGG |
| CREB5-Enhancer-sgRNA-3 | CGGAGTGCTGTGTTTCGGCCA |
