# Supplementary material for: Endoplasmic reticulum stress-related super enhancer promotes epithelial-mesenchymal transformation in hepatocellular carcinoma through CREB5 mediated activation of TNC
Source: Cell Death Dis. 2025 Feb 6;16(1):73. doi: 10.1038/s41419-025-07356-y (PMC11802765; doi:10.1038/s41419-025-07356-y)
Supplement: Supplementary file 12 — Supplementary Table 7 [file 41419_2025_7356_MOESM12_ESM.docx]

**Table S7. Sequence of primers for Luciferase reporter assay and ChIP-qPCR.**

| primer | Sequence (5’to 3’) |
| --- | --- |
| TNC-promoter(-2000 ~ +30)-F | CGGTACCTGAGCTCGCTAGCTTTGAGACATTCCCCGAAAAAG |
| TNC-promoter(-2000 ~ +30)-R | AGTACCGGATTGCCAAGCTTGAGGCGGGTGACAGTAGGCAGGC |
| TNC site1 F | TTGAGACACAGCTTCATGCTAC |
| TNC site1 R | CCTCCCAGACAGCAAGAAAG |
| TNC site2 F | TGGGAGGGTCACTTTAGCAG |
| TNC site2 R | CCTCAGTTTCCCCATCTGAA |
| TNC site3 F | AGGCCCACAGCAACAATAAT |
| TNC site3 R | TTGCTCCATAAAGCTAATTAGGAGA |
| GAPDH-F | AAAAGCGGGGAGAAAGTAGG |
| GAPDH-R | AAGAAGATGCGGCTGACTGT |
