# Supplementary material for: Endoplasmic reticulum stress-related super enhancer promotes epithelial-mesenchymal transformation in hepatocellular carcinoma through CREB5 mediated activation of TNC
Source: Cell Death Dis. 2025 Feb 6;16(1):73. doi: 10.1038/s41419-025-07356-y (PMC11802765; doi:10.1038/s41419-025-07356-y)

Figure 1E Raw image

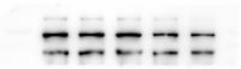

BRD4

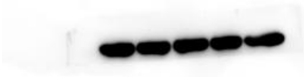

β-actin

Figure 1G Raw image

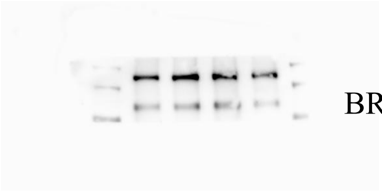

BRD4

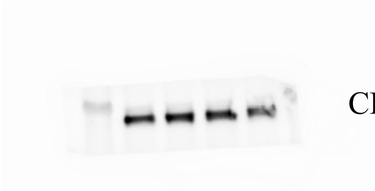

CREB5

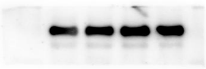

β-actin

Figure 2M Raw image

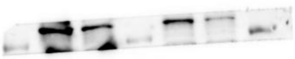

CREB5

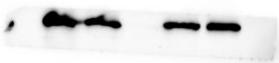

β-actin

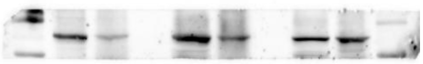

CREB5

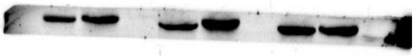

β-actin

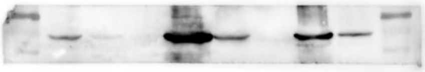

CREB5

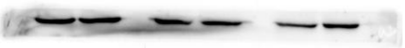

β-actin

Figure 3A Raw image

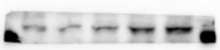

CREB5

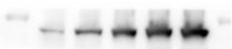

GRP78

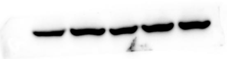

β-actin

Figure 3C Raw image

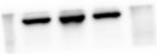

CREB5

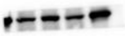

GRP78

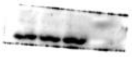

β-actin

Figure 5I Raw image

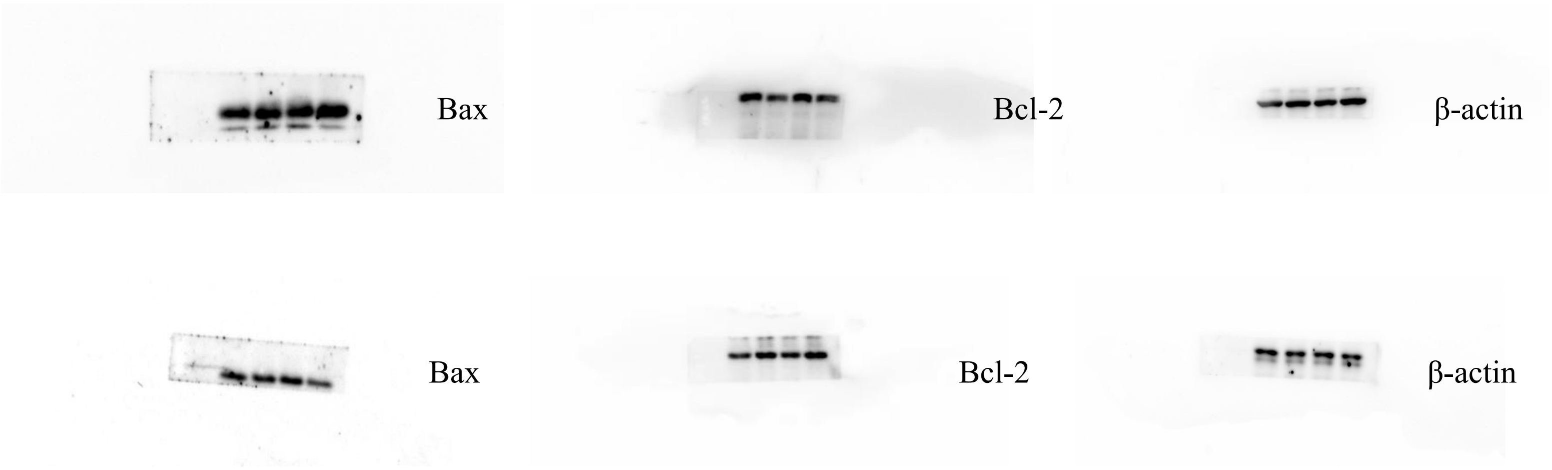

Figure 6B Raw image

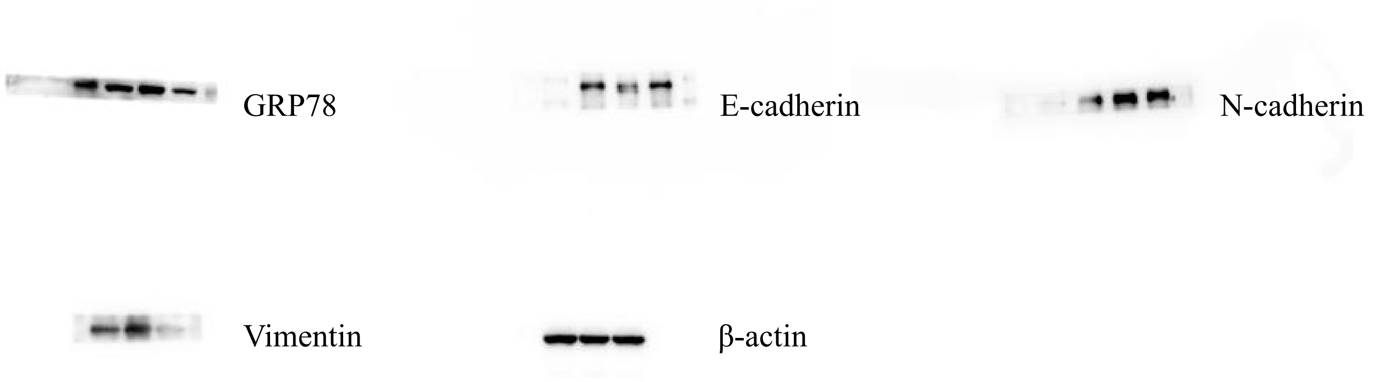

Figure 6H Raw image

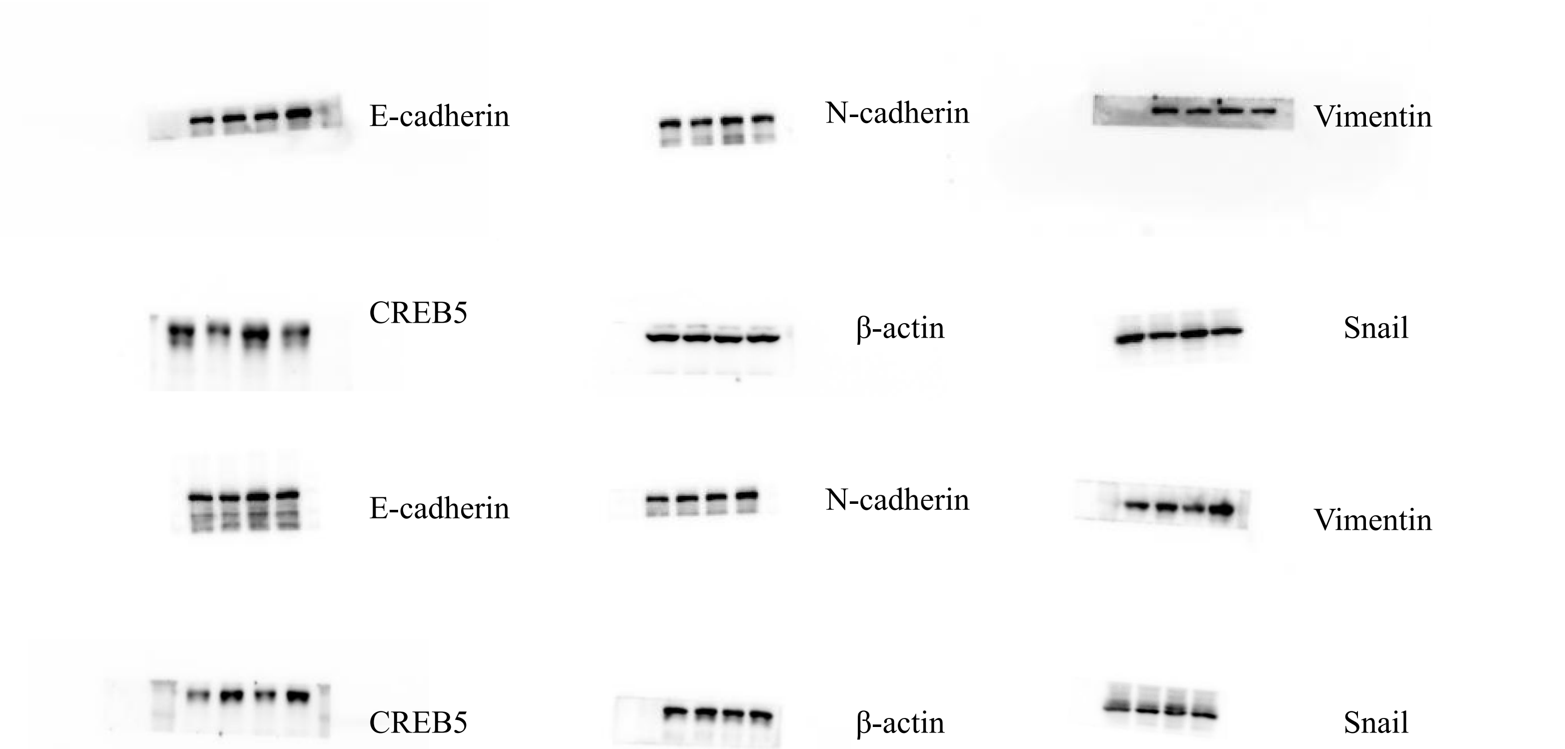

Figure 6I Raw image

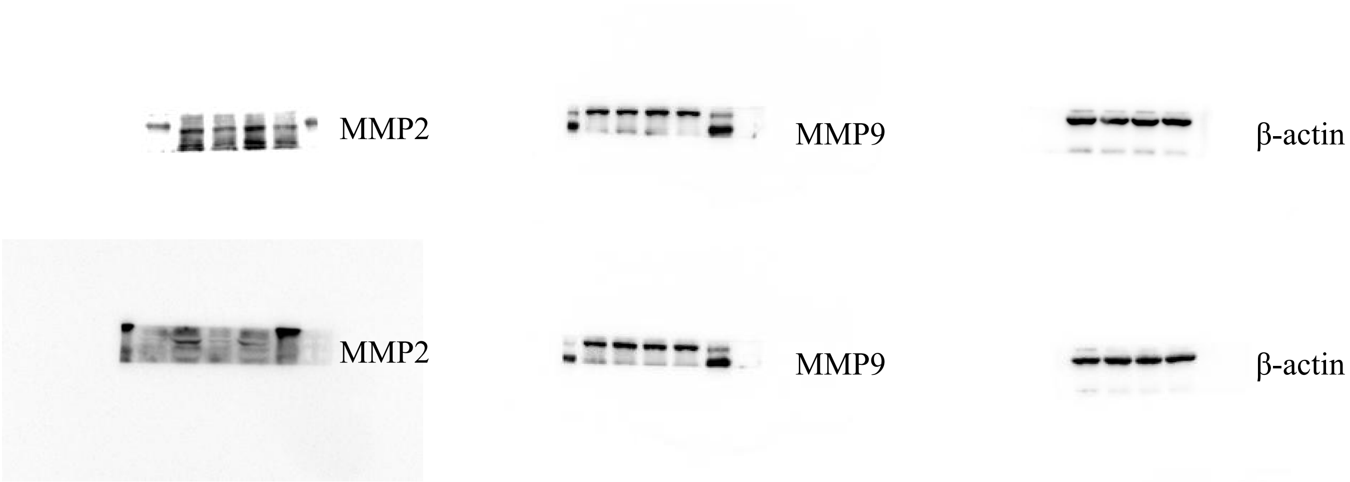

Figure 7D Raw image

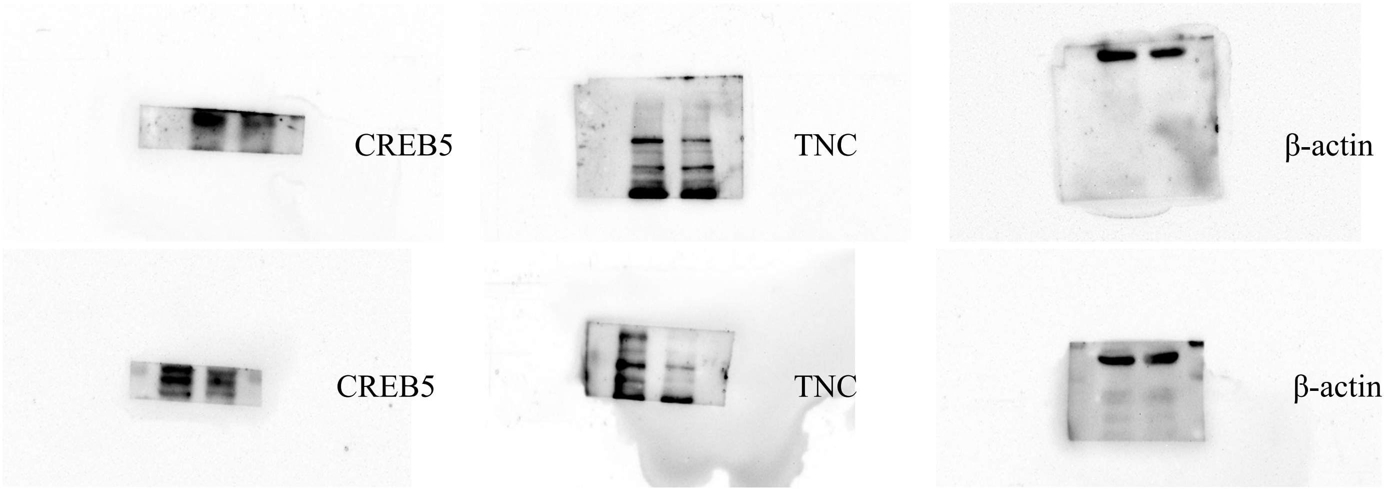

Figure 7E Raw image

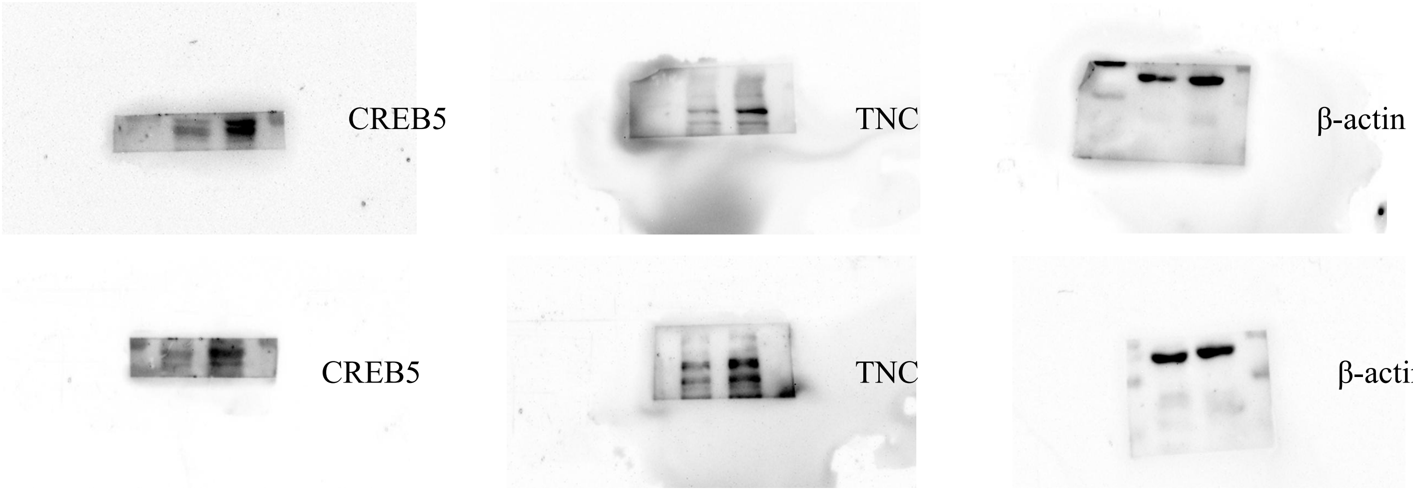

Figure 8B Raw image

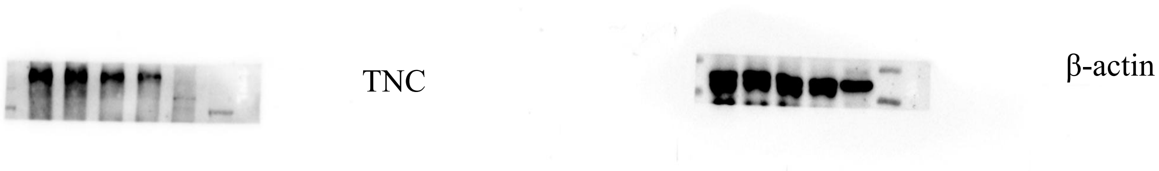

Figure 8E Raw image

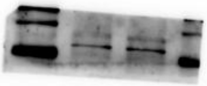
E-cadherin

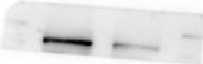
N-cadherin

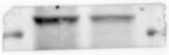
Vimentin

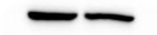
β-actin

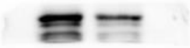
Snail

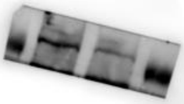
MMP2

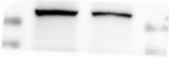
MMP9

Figure 8H Raw image

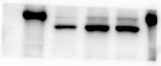
CREB5

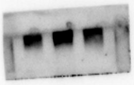
TNC

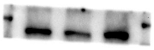
E-cadherin

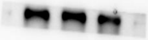
N-cadherin

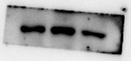
Vimentin

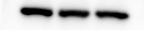
β-actin

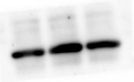
Snail

Supplementary Figure 1E Raw image

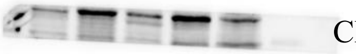
CREB5

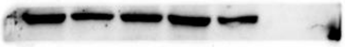
β-actin

Supplementary Figure 2A Raw image

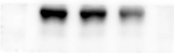
CREB5

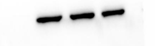
β-actin

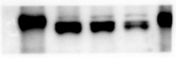
CREB5

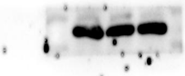
β-actin

Supplementary Figure 2F Raw image

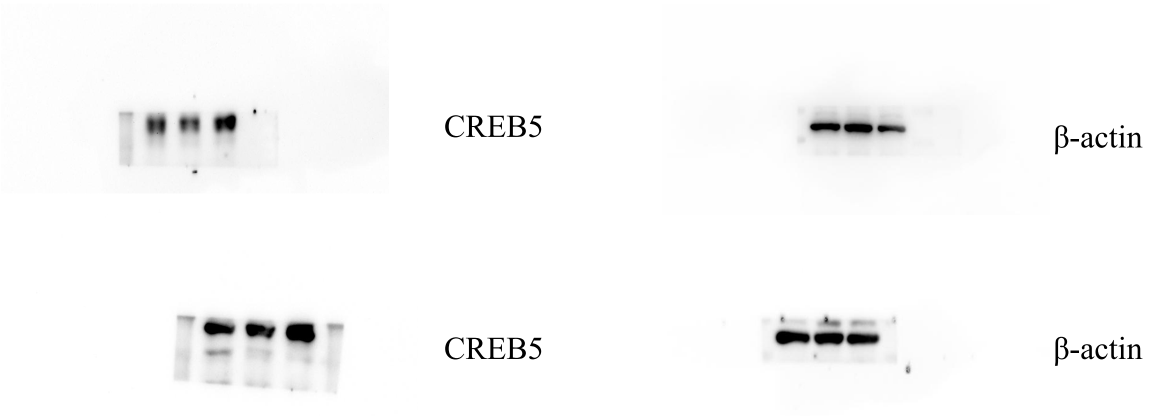

Supplementary Figure 3F Raw image

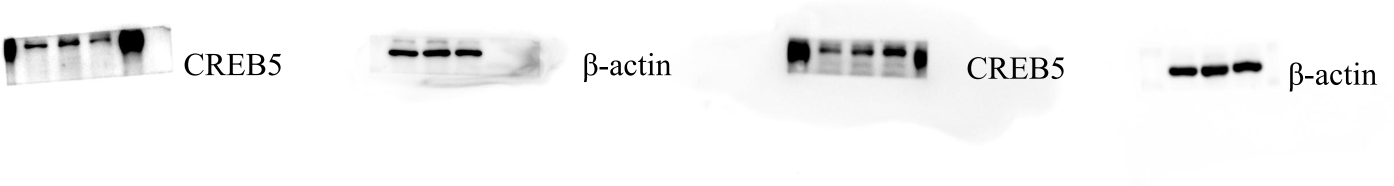

Supplementary Figure 3H Raw image

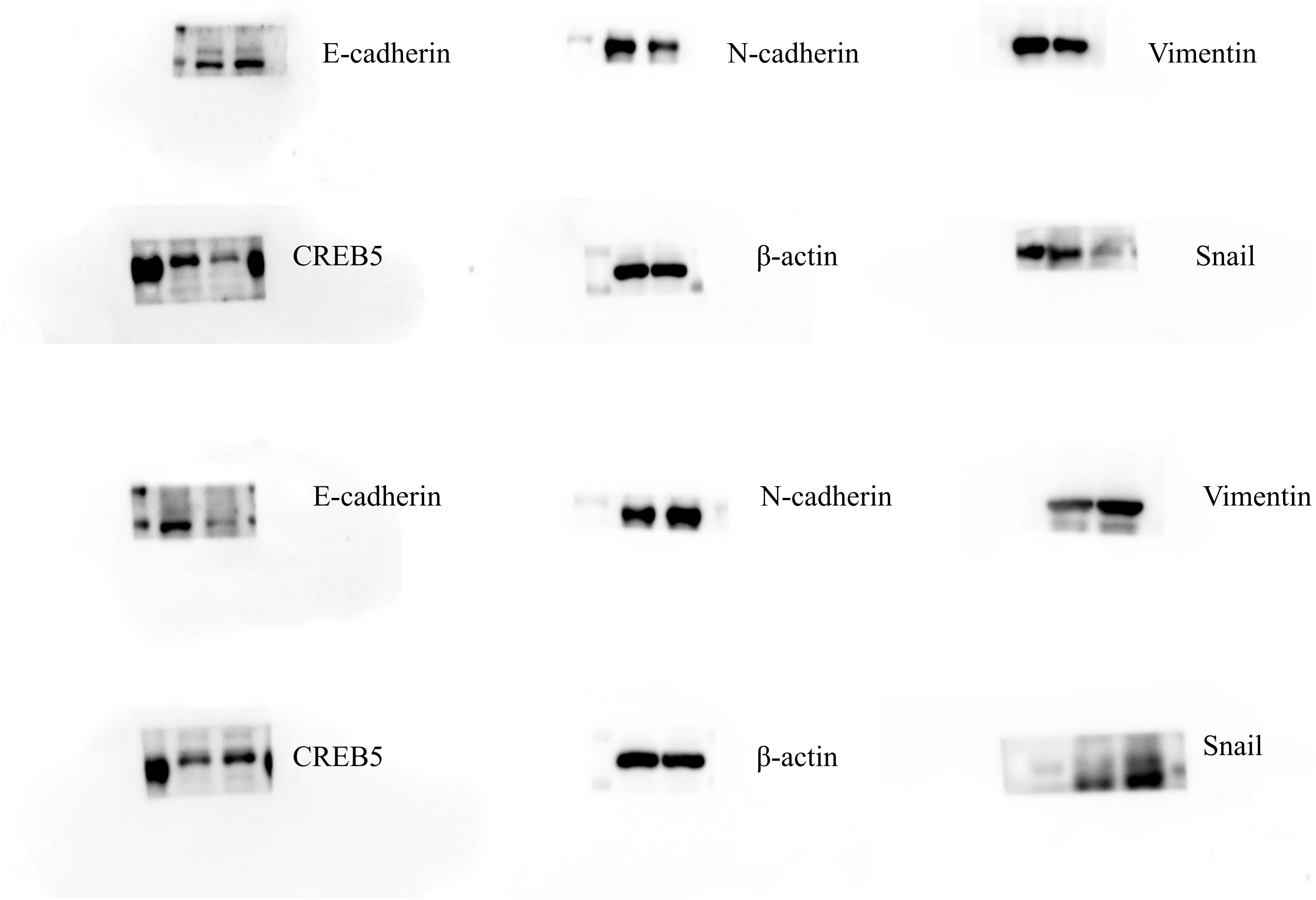

Supplementary Figure 3J Raw image

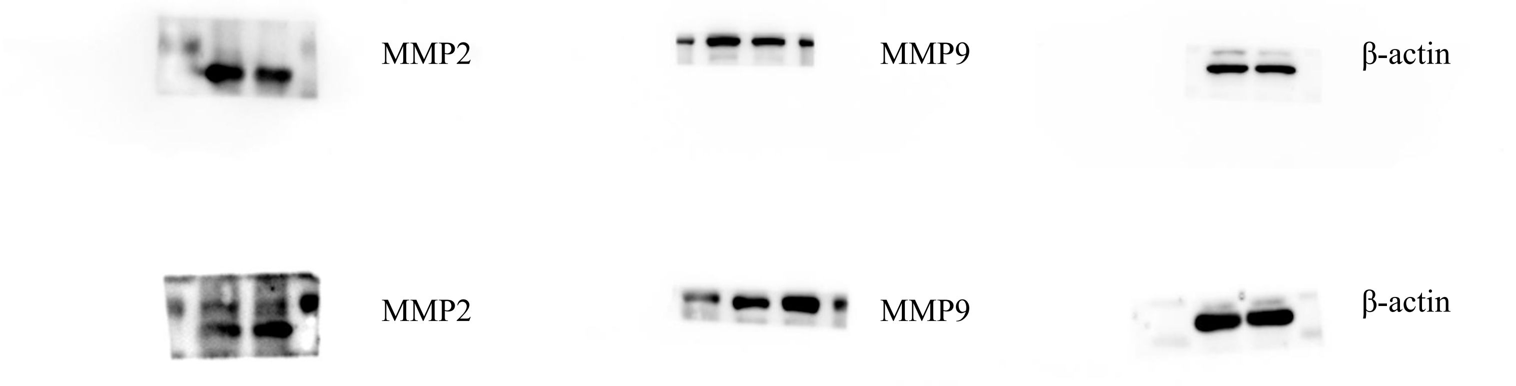

Supplementary Figure 4E Raw image

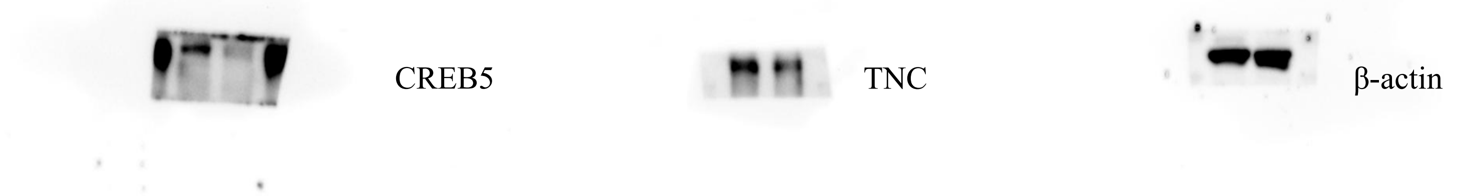

Supplementary Figure 4F Raw image

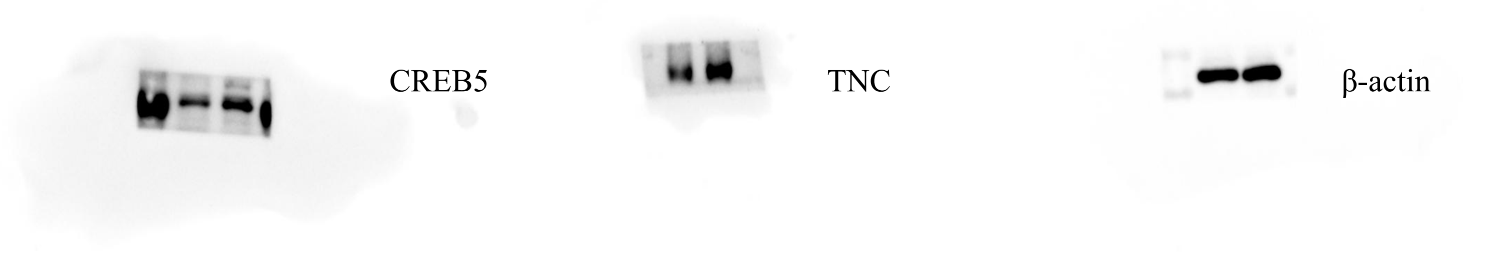

Supplementary Figure 4I Raw image

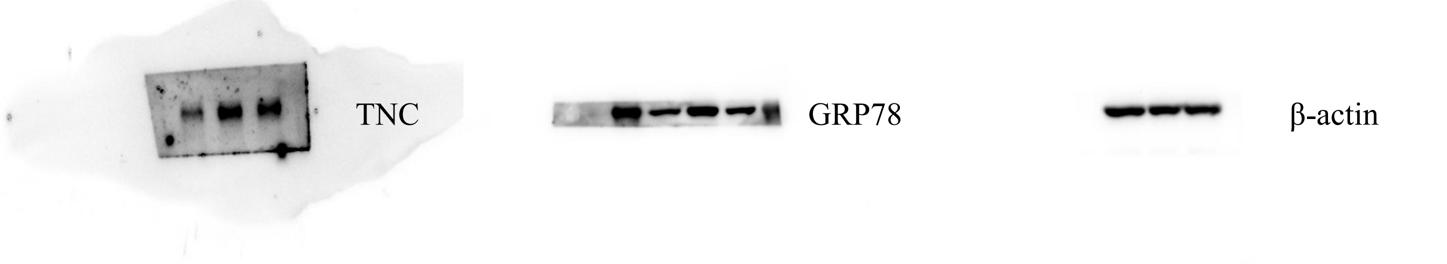

Supplement: Supplementary file 13 — Original western blots [file 41419_2025_7356_MOESM13_ESM.pdf]
